# Large‐scale genome‐wide association study, using historical data, identifies conserved genetic architecture of cyanogenic glucoside content in cassava (*Manihot esculenta* Crantz) root

**DOI:** 10.1111/tpj.15071

**Published:** 2020-12-18

**Authors:** Alex C. Ogbonna, Luciano Rogerio Braatz de Andrade, Ismail Y. Rabbi, Lukas A. Mueller, Eder Jorge de Oliveira, Guillaume J. Bauchet

**Affiliations:** ^1^ Cornell University 135 Plant Science Building Ithaca NY 14850 USA; ^2^ Boyce Thompson Institute 533 Tower Rd Ithaca NY 14853 USA; ^3^ Embrapa Mandioca e Fruticultura Rua Embrapa s/nº, Caixa Postal 007 Cruz das Almas BA CEP: 44380‐000 Brazil; ^4^ International Institute of Tropical Agriculture PMB 5320, Oyo Road Ibadan Oyo State 200001 Nigeria

**Keywords:** genetic architecture, epistasis interaction, domestication, phylogenetics, cyanogenic glucosides, *Manihot esculenta* Crantz, MATE transporter

## Abstract

*Manihot esculenta* (cassava) is a root crop originating from South America that is a major staple in the tropics, including in marginal environments. This study focused on South American and African germplasm and investigated the genetic architecture of hydrogen cyanide (HCN), a major component of root quality. HCN, representing total cyanogenic glucosides, is a plant defense component against herbivory but is also toxic for human consumption. We genotyped 3354 landraces and modern breeding lines originating from 26 Brazilian states and 1389 individuals were phenotypically characterized across multi‐year trials for HCN. All plant material was subjected to high‐density genotyping using genotyping by sequencing. We performed genome‐wide association mapping to characterize the genetic architecture and gene mapping of HCN. Field experiments revealed strong broad‐ and narrow‐sense trait heritability (0.82 and 0.41, respectively). Two major loci were identified, encoding for an ATPase and a MATE protein, and contributing up to 7 and 30% of the HCN concentration in roots, respectively. We developed diagnostic markers for breeding applications, validated trait architecture consistency in African germplasm and investigated further evidence for the domestication of sweet and bitter cassava. Fine genomic characterization revealed: (i) the major role played by vacuolar transporters in regulating HCN content; (ii) the co‐domestication of sweet and bitter cassava major alleles are dependent upon geographical zone; and (iii) the major loci allele for high HCN in *M. esculenta* Crantz seems to originate from its ancestor, *M. esculenta* subsp*. flabellifolia*. Taken together, these findings expand our insights into cyanogenic glucosides in cassava roots and its glycosylated derivatives in plants.

## Significance Statement

The high cyanogenic glucoside content in some cassava varieties prevents herbivory but can be toxic for human consumption. The identification of an intracellular transporter gene and its allelic variation allow us to identify cultivars with up to 30% reduced cyanogenic glucoside content in cassava root.

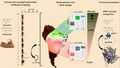

## INTRODUCTION

*Manihot esculenta* Crantz (cassava) is a starchy root crop that is widely grown throughout the tropics (in Southeast Asia, Latin America, the Caribbean and sub‐Saharan Africa) for human and livestock consumption, and as feedstock for biofuels and other bio‐based materials (Fregene and Puonti‐Kaerlas, 2002; Howeler *et al*., 2013). Mostly cultivated by low‐income smallholder farmers, cassava is a staple food crop for over 800 million people worldwide. Cassava is an efficient crop in marginal areas where poor soils and unpredictable rainfall dominate (Howeler, 2013). All plants produce tiny quantities of hydrogen cyanide (HCN) as an additional product in the biosynthesis of ethylene, but some plant species can release large quantities of HCN from endogenously stored cyanogenic glycosides (McKey and Beckerman, 1993; Gleadow and Møller, 2014). Cassava has developed defense mechanisms against herbivores and pathogens, including the biosynthesis of cyanogenic glucosides (CGs) (Nordenskiold, 1924; De Bruijn, 1973; McKey and Beckerman, 1993; Tattersall *et al*., 2001; Zagrobelny *et al*., 2004; Gleadow and Møller, 2014); however, some of the major challenges in cassava include low tuber protein and carotenoid content as well as the high content of CGs (Jørgensen *et al*., 2005; Blomstedt *et al*., 2012; Gleadow and Møller, 2014). CGs, characterized as α‐hydroxynitriles, are secondary metabolites derived from amino acids (Gleadow and Møller, 2014). Cyanogenesis occurs when CGs release toxic HCN in cassava roots upon tissue disruption. HCN concentrations are usually higher in young plants, when nitrogen is in ready supply, or when growth is constrained by non‐optimal growth conditions (Gleadow and Møller, 2014).

Cyanogenic glucosides (CGs) are assayed as the HCN trait, a proxy representing total CGs (HCN/CN^–^, linamarin and acetone cyanohydrin) (Bradbury *et al*., 1999; Fukuda *et al*., 2010). Cultivars with HCN contents of <100 mg kg^−1^ fresh weight (FW) are called ‘sweet cassava’, whereas cultivars with 100–500 mg kg^−1^ FW are called ‘bitter cassava’ (Wheatly *et al*., 2003). In Brazil, the center of diversity for cassava, the preference for bitter or sweet cassava appears to be linked with its role in subsistence farming in the regions where that type of cassava dominates. In regions where the sweet cassava type dominates, it is a component of a diet in which *Zea mays* (maize) is more important; whereas in regions where the bitter cassava type dominates, it is the main carbohydrate source, generally complemented by a protein, such as a fish (Mühlen *et al*., 2019).

Cyanogenic glucosides (CGs) in cassava are synthesized in the leaves and then transported to the roots via the phloem (Jørgensen *et al*., 2005). Linamarin and lotaustralin are the two main forms of CG in cassava (Santana *et al*., 2002), but the most abundant CG is linamarin (representing 95% of CGs) (Padmaja and Steinkraus, 1995), and total CG concentration varies according to the cultivar, environmental conditions, cultural practices and plant age (McMahon *et al*., 1995). The degradation of linamarin is catalyzed by the enzyme linamarase, which is found in cassava tissues, including intact roots. The compartmentalization of linamarase in cell walls and linamarin in vacuoles prevents the accidental formation of free HCN. Disruption of these tissues ensures that the enzyme comes into contact with its substrate, resulting in the rapid production of free HCN via an unstable cyanohydrin intermediary (Wheatly *et al*., 2003). Therefore, careful processing is required to remove HCN, especially in communities with poor nutritional status (Jørgensen *et al*., 2005; Blomstedt *et al*., 2012; Gleadow and Møller, 2014). Incomplete processing could result in acute or chronic exposure to HCN (Leavesley *et al*., 2008). High dietary cyanogen consumption from insufficiently processed roots of bitter cassava combined with a protein‐deficient diet leads to a neglected disease known as konzo (Kashala‐Abotnes *et al*., 2019). Konzo is a distinct neurological disease characterized by the abrupt onset of an irreversible, non‐progressive paralysis of the limbs (Tshala‐Katumbay *et al*., 2001; Nzwalo and Cliff, 2011; Kashala‐Abotnes *et al*., 2019). Juice extraction, heating, fermentation, drying or a combination of these processing treatments aid in reducing the concentration of HCN to safe levels (Wheatly *et al*., 2003). Gleadow and Møller (2014) reported efforts in cassava breeding programs to actively select for varieties with lower levels of HCN; however, some farmers favor cassava varieties with higher HCN contents as a source of resistance against herbivores and theft by humans (McKey and Neckerman, 1993; Lebot, 2009). Modern breeding has not yet succeeded in developing cassava cultivars that are totally free of CGs (Nweke *et al*., 2002; Jørgensen *et al*., 2005). Previous studies (Kizito *et al*., 2007; Whankaew *et al*., 2011) on HCN, using a quantitative trait locus (QTL) approach, could not provide conclusive information on the genetic basis for HCN variation in cassava, owing to the genomic resources and narrow data set available so far.

In this study, we seek to: (i) comprehensively understand the genetic architecture of the HCN trait (total CGs) in cassava root; (ii) map the gene(s) associated with CG variation; (iii) develop a fast and cost‐effective molecular diagnostic toolkit for breeding purposes to increase selection efficiency; and (iv) investigate the role of HCN in domestication.

## RESULTS

### Large‐scale analysis of Brazilian population for HCN content

**Figure 1:**
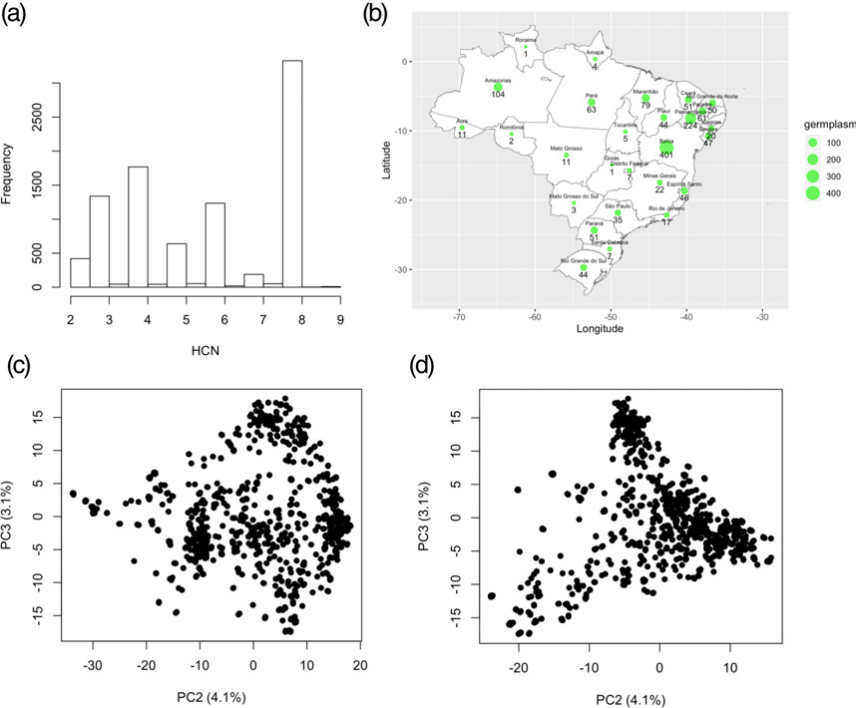
Geographic distribution and population structure of Latin American (Brazilian) germplasm. (a) Distribution of assayed HCN. HCN assayed phenotype score ranges from 2 to 9. (b) Distribution of germplasm based Brazilian states. A total of 1,821 cassava accessions have valid geographic information. The green dots show where the accessions come from while the size of the dots represent the number of clones from that state. The black numbers show how many accessions were sampled from each location. Population structure reveals the first three axis of the principal component analysis (PCA) explains about 15.3% of the variations in the population of 1246 individuals, 9,686 SNPs after filtering for Hardy–Weinberg equilibrium and LD of 0.01 and 0.2 respectively. (c) shows the first and third axis, while (d) shows the second and the third axis.

Phenotypic distribution and variation for HCN content was measured in a Brazilian population of 1246 individuals using the picrate titration method, in which a scale of 1–9 indicates the concentration of HCN content (with 1 and 9 representing extremes of low and high HCN concentration, respectively) (Bradbury *et al*., 1999; Fukuda *et al*., 2010). Based on an empirically determined scale the HCN concentration varies from 2 to 9, with an average value of 5.6 in samples from across Brazilian states (Figure 1a,b). About two‐thirds of the 28 203 total plots had missing values, with 9139 plots having HCN observations (Tables S1 and S2). Broad‐sense heritability (*H* ^2^) was calculated as 0.82 for HCN content, similar to previous observations reported on several species (Barnett and Caviness, 1968; Goodger *et al*., 2004; Gleadow and Møller, 2014). Using genotyping data previously recorded for this population (Ogbonna *et al*., 2020), we observed genotype variance (*V*_g_) that was higher than genotype‐by‐year variance (*V*_g × y_), with the *V*_g × y_/*V*_g_ ratio showing a year interaction value of 0.29. HCN‐deregressed best linear unbiased prediction (BLUP) shows a very high correlation with non‐deregressed BLUP, with a Pearson’s correlation coefficient of 0.99, indicating a balanced replication of individuals among the population studied (see deregressed BLUPs, Table S3).

### Genome‐wide association study (GWAS) analysis revealed two SNPs associated with HCN accumulation

Single‐nucleotide polymorphisms (SNPs) calling in tassel 5 identified a total of 343 707 variants, 30 279 of which were selected for phasing and imputation. After imputation, a total of 27 045 biallelic SNPs with an allelic correlation of 0.8 or above were kept for downstream analysis. The first three principal components (PCs) accounted for over 15.3% of the genetic variation (Figure 1c,d; Appendix S1).

**Figure 2:**
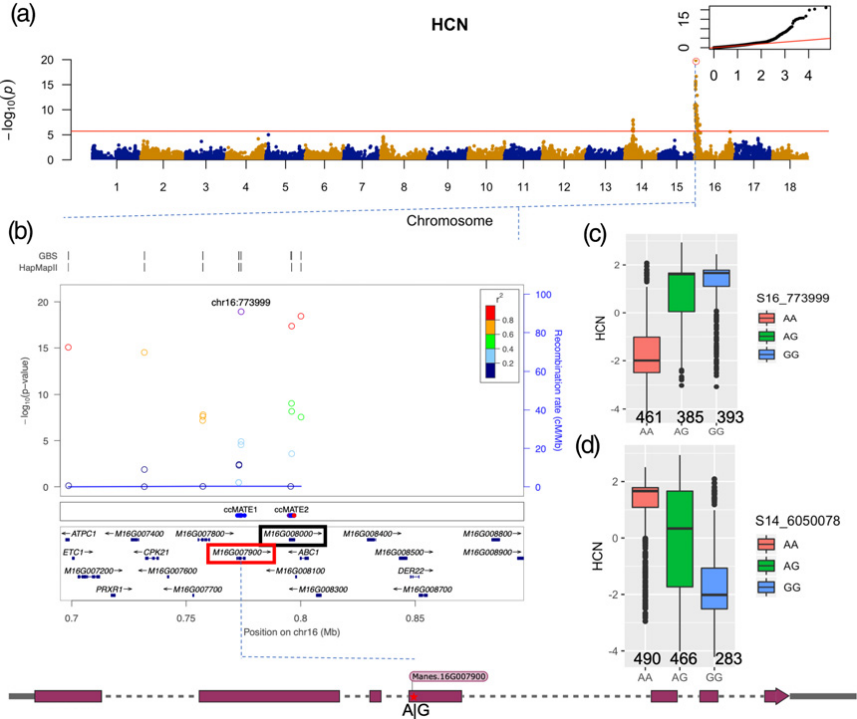
Genome‐wide association study (GWAS) of HCN for Latin American (LA) germplasm. (a) Manhattan plot from a mixed linear model (MLM‐LOCO) with the chromosome on which the candidate SNP is located excluded from calculating the genetic relationship matrix (GRM). The Bonferroni significance threshold is shown in red. A quantile–quantile plot is inserted to demonstrate the observed and expected −log_10_ *P* for HCN. The red circle indicates the candidate SNP. (b) locuszoom plot showing the HCN chromosome 16‐associated region (−log_10_ *P*) around the candidate gene. The two rows above the plot show genomic coverage at the locus, with each vertical tick representing direct genotyping from GBS and HapMap single‐nucleotide polymorphisms (SNPs). Each circle represents an SNP, with the color of the circle indicating the correlation between that SNP and the candidate SNP at the locus (purple). Light‐blue lines indicate the estimated recombination rate (hot spots) in GBS. The middle panel shows 36 single point mutations (red are deleterious) between the region spanning ccMATE1 and ccMATE2. The bottom panel shows the annotated genes at each locus in cassava genome version 6.1. The red and black rectangles indicate *Manes.16G007900* and *Manes.16G008000*, respectively, with a Pearson correlation coefficient of 0.96 (*r* ^2^) between both genes. The scheme presents the gene model, with the position of the associated SNP within the 4th exon indicated. (c and d) Box plots showing candidate SNP effects for HCN between each genotype class for the top markers, S14_6050078 and S16_773999, respectively.

To identify genetic correlation between HCN content and genotypic variation, mixed‐model GWAS was performed using gcta (Yang *et al*., 2011), with Bonferroni correction as a test of significant SNPs. After Bonferroni correction, with a −log_10_(0.05/27045) threshold of 5.733117, two significant peaks were identified on chromosomes 14 and 16, with 45 and 12 significant associated markers, respectively (Figure 2a; Table S4). Subsequent regional linkage disequilibrium (LD) analysis on chromosome 16 gives a 3.6‐Mb interval and local LD analysis gives a 248‐Kb interval (with an *r* ^2^ threshold of >0.8) in which six genes are annotated (Figure S1a; Tables 1 and 2). The optimal strongest *P* value indicates the SNP S16_773999 (*P* = 7.53E–22) is located within the *Manes.16G007900* gene. *Manes.16G007900* is annotated as a multidrug and toxic compound extrusion or multi‐antimicrobial extrusion (MATE) protein. MATE transporters are a universal gene family of membrane effluxers present in all kingdoms of life. MATE transporters have been implicated directly or indirectly in the mechanisms of detoxification of noxious compounds and are able to transport CGs (Darbani *et al*., 2016). Interestingly, the S16_773999 SNP is predicted to induce a missense variant (A to G) in exon 4 (Figure 2b, marked with a red star in the gene model). This mutation causes an amino acid change from Thr to Ala, and is predicted to be deleterious. A second MATE gene (*Manes.16G008000*) located 22 Kb from the candidate MATE gene (Figure 2b, annotation panel) also shows a high LD (pairwise correlation of 0.96; Figure S2a). The second MATE gene could be a paralog of the *Manes.16G007900* gene from a tandem duplication event, a frequent phenomenon observed in the MATE gene family (Cannon *et al*., 2004; Santos *et al*., 2017).

**Table 1:** Summary of linkage disequilibrium (LD) analysis within the regions (chromosomes 14 and 16) associated with HCN variation in *Manihot esculenta* (cassava) for Brazilian germplasm

Chromosome	GWAS LD intervals	High LD intervals (*r* ^2^ > 0.8)	Main candidate gene	Annotation
16	3.6 Mb (409 genes)	248 Kb (6 genes) 658 264–800 090 bp	Transporter	Multidrug and toxic compound extrusion
14	615 Kb (77 genes)	274 Kb (3 genes) 5 775 892–6 070 331 bp	ATPase protein	Plasma membrane H^+^‐ATPase

**Table 2:** Summary of genes within the regions associated with HCN variation in Brazilian *Manihot esculenta* (cassava)

Chromosome	SNP	Position (bp)	Allele	*P*	Gene	Name	Function	Reference
16	S16_773999	773 999	G/A	7.53E–22	*Manes.16G007900*	MATE efflux family protein	Multidrug and toxic compound extrusion	Darbani *et al*., 2016
16	S16_795990	795 990	A/T	2.41E–10	*Manes.16G008000*	MATE efflux family protein	Multidrug and toxic compound extrusion	
16	S16_796041	796 041	T/A	1.36E–20	*Manes.16G008100*			
16	S16_800090	800 090	A/T	4.33E–21	*Manes.16G008200*	UPF0051 PROTEIN ABCI8, CHLOROPLASTIC‐RELATED	The incorporation of iron and exogenous sulfur into a metallo‐sulfur cluster	
16	S16_698521	698 521	A/G	2.08E–16	*Manes.16G007000*	F‐type H^+^‐transporting ATPase subunit gamma (ATPF1G, atpG) (ATP synthase)	The sector of a hydrogen‐transporting ATP synthase complex in which the catalytic activity resides	
16	S16_658264	658 264	T/C	2.34E–16	*Manes.16G006300*	ANKYRIN REPEATFAMILY PROTEIN		
14	S14_6050078	6 050 078	G/A	1.09E–08	*Manes.14G074300*	Integral membrane HPP family protein	Involved in nitrite transport activity	Maeda *et al*., 2014
14	S14_5775892	5 775 892	G/T	1.63E–08	*Manes.14G071000*	K03355 anaphase‐promoting complex subunit 8 (APC8, CDC23)	Interacting selectively and non‐covalently with any protein or protein complex	
14	S14_6021712	6 021 712	A/T	7.32E‐08	Manes.14G073900	H(+)‐ATPase (The plasma membrane H+‐ATPase)	Associated with the plasma membrane gradients coupled to the MATE co‐transport system	Zhang *et al*., 2017 and Wu *et al*., 2014

The second peak in chromosome 14 shows an association with a log_10_ *P* value of 1.08e–08 and associated interval of 615 Kb; local LD analysis reduced this interval to 274 Kb, where three genes are located (Figure S1b; Tables 1 and 2). The first candidate SNP indicates that S14_6050078 (*P* = 1.08E–08) is located in *Manes.14G074300*, a gene coding for an integral membrane HPP family protein involved in nitrite transport activity (Maeda *et al*., 2014). In a recent study, Obata *et al*. (2020) highlighted that linamarin, an abundant CG variant in cassava, contains nitrogen and serves as a nitrogen storage compound (Obata *et al*., 2020), as previously hypothesized (Siritunga and Sayre, 2004). This is congruent with previous observations that the application of nitrate fertilizer to cassava plants increases CG accumulation in the shoot apex (Jørgensen *et al*., 2005). The second candidate SNP indicates that S14_6021712 (*P* = 7.32E–08) is located in *Manes.14G073900.1*, coding for a plasma membrane H^+^‐ATPase. H^+^‐ATPase mediates the influx of H^+^ associated with aluminimum (Al)‐induced citrate efflux coupled with a MATE co‐transport system (Zhang *et al*., 2017). Wu *et al*. (2014) found that transgenic Arabidopsis lines containing a *Brassica oleracea* MATE gene had stronger citrate exudation coupled with higher H^+^ efflux activity than wild‐type plants (Wu *et al*., 2014).

**Figure 3:**
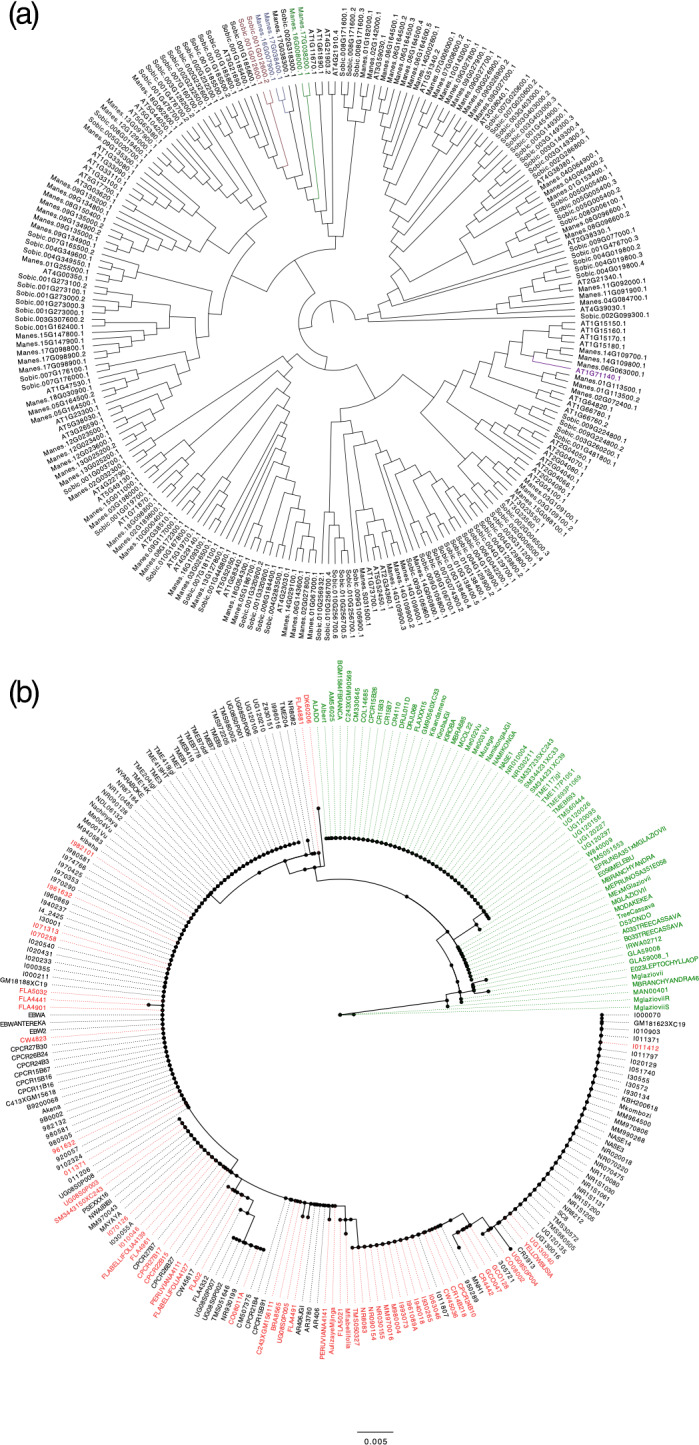
Phylogeny analysis. (a) Protein sequences alignment of MATE genes in cassava, sorghum and Arabidopsis. Protein alignment and comparative phylogeny show a close sequence homology between the genome‐wide association study (GWAS) candidate gene and SbMATE2 (Sobic.001G012600), a vacuolar membrane MATE transporter characterized in sorghum that functions in the accumulation of specialized plant metabolites, such as flavonoids and alkaloids. (b) Proteic sequence of Manes.16G007900 for the 242 HapMap accessions. Accessions highlighted in red are homozygous G:G for SNP16_773999, identified as having high HCN content. Accessions highlighted in green are homozygous A:A for SNP16_773999, identified as low HCN content. Accessions in black are heterozygotes A:G or G:A for SNP16_773999. [Correction added on 8 January 2021, after first online publication: The legends of Figure 3 and 4 were swapped in the original publication; this has been amended.]

As a validation step, we used a subset of 523 unique individuals (from the core panel of 1536 unique individuals; Ogbonna *et al*., 2020) with phenotypic and genotypic information to perform GWAS. Our results (Figure 3, Unique HCN; Table S5) revealed the same loci (as was observed in the larger data set of 1246 individuals) associated with HCN variation in our initial GWAS data set, indicating that the core unique panel represents the overall genetic variation for HCN in the Brazilian germplasm collection. GWAS detected less significant loci (only 46%) than those detected using a data set of 1246 individuals, however. This indicates that additional small‐effect QTLs were captured with the larger data set through increased statistical power.

The alleles driving high HCN at S16_773999 and S14_6050078 loci show dominance and additive patterns, respectively (Figure 2c,d); homozygotes with alternate alleles for both loci show higher HCN content than heterozygotes, whereas homozygotes with reference alleles show lower HCN content. This indicates that cyanogenic cassava can either be homozygous or heterozygous for alleles at these loci, whereas acyanogenic cassava plants are more likely to be homozygous for a reference allele at these loci. Joint allelic substitution effects at the associated loci for HCN did not show any interaction between the two loci, as shown in Figure S1(c).

### Variance explained and evidence for domestication in HCN reveals chromosome 16 as a good candidate for Kompetitive Allele Specific PCR (KASP) marker development

To calculate narrow‐sense heritability, the proportion of variance explained was calculated using a parametric mixed model multiple kernel approach (Akdemir and Jannink, 2015). A single‐kernel mixed model explained 0.41 of the marker‐based proportion of the variance for HCN across the genome (narrow‐sense heritability, *h* ^2^). A multi‐kernel mixed model with the top significant SNPs in chromosomes 16 and 14 (S16_773999 and S14_5775892) as the first and second kernels, with the rest of the genome as the third kernel, explained 30, 7 and 63% of the marker‐based variance, respectively. A three‐kernel mixed model to determine the variance explained by chromosomes 14, 16 and the rest of the genome showed that the proportion of variance explained by the three kernels are 16, 50 and 34%, respectively. Chromosomes 14 and 16 tagging SNPs for the candidate SNPs explains 8 and 36% proportions of variance, respectively, whereas the rest of the genome explains 56% of the variance. We found evidence for local interactions within chromosome 16, most likely as a result of high LD around the region (Methods S1).

To validate the local interaction found in chromosome 16, we performed an intrachromosomal epistasis interaction using factored spectrally transformed linear mixed models (FaST‐LMMs) (Lippert *et al*., 2011, 2013). Chromosome 16 revealed 242 significant interactions above the Bonferroni‐corrected threshold of −log_10_(0.05/1131*(1131 – 1)/2) = 1.6024, with three interactions clearly separated by 1 Mb between each pair of SNPs (Figure S1d; Tables 3 and Table S9). A biosynthetic gene cluster in cassava (genome version 4.1) was identified previously by Andersen *et al*. (2000) and Takos *et al*. (2011), which we identified to be present on chromosome 12 in genome version 6.1, as shown in Figure S3(a,b). Interchromosomal epistasis interaction analysis, involving about 400 million tests, did not reveal any significant interactions using either a Bonferroni or a false discovery rate (FDR) threshold. Over 27 million tests had *P* values that were less than the 0.05 significance level (Methods S2).

**Table 3:** Chromosome 16 single‐nucleotide polymorphism (SNP) pairs for separated (1 Mb apart) epistasis Interactions in chromosome 16. SNP1 and SNP2 showed strong significant interactions. The table also contains single‐locus *P* values for the interaction SNPs (SNP)

SNP 1		SNP 2		Interactions
SNP	Single‐locus *P* value	SNP	Single‐locus*P* value	Interaction *P* value
S16_12540	1.00E–05	S16_1063230	4.31E–15	5.207858e–10
S16_1298874	2.01E–07	S16_12540	1.00E–05	2.969058e–09
S16_1298876	7.72E–08	S16_12540	1.00E–05	8.371752e–10

Investigating the evidence for domestication in HCN, we carried out differentiating loci analysis using cassava HapMap reference lines (Ramu *et al*., 2017) for cultivated *M. esculenta* and wild *M. esculenta* subsp*. flabellifolia* (Table S6). We identified 294 biallelic ancestry‐informative SNPs that represent fixed or nearly fixed differences between cultivated and wild accessions (Figure S4). Interestingly, we observed a high number of fixed loci (89) differentiating between the two groups in chromosome 16, over 54 of which are approximately 0.37 Mb away from the candidate MATE gene for HCN regulation (Figure S4). Together, these results indicate that: (i) epistasis is observed within chromosome 16 around the main GWAS peak (Figure S1d); and (ii) the epistatic region identified colocalizes with differentiating loci between *M. esculenta* and wild *M. esculenta* subsp*. flabellifolia* (Methods S3; Table S6).

The KASP assay is robust, high‐throughput and cost‐effective PCR‐based marker technology (Neelam *et al*., 2013; He *et al*., 2014). We used KASP to develop and validate diagnostic markers for HCN content, based on association peaks, local LD and allelic effects. Candidate SNPs from the GWAS were subjected to KASP marker design (Table S7) and then assayed on Embrapa Breeding populations for a total of 576 individuals. The average percentage genotype score or call rate was 96.59%, with a maximum of 97.92% and a minimum of 92.71% validated allelic segregation for HCN content (Methods S4; Table S8).

### Phylogenetics and mutation predictions reveal altered function of MATE transporter

To identify homologs of the MATE transporter Manes.16G007900, protein alignment and comparative phylogeny analysis was performed for genome‐wide MATEs in cassava, sorghum and Arabidopsis using clustal omega (Sievers *et al*., 2011). Results showed a close sequence homology between three additional MATE transporters in the cassava genome: *Manes.16G007900*, *Manes.17G038400*, *Manes.17G038300* and *Manes.16G00800*, with percentage identities of 91.09, 78.05 and 68.59%, respectively. The highest interspecific homology was found for SbMATE2 from sorghum (*Sobic.001G012600*; percentage identity of 67.84% for first isoform and 71.00% for second isoform) (Darbani *et al*., 2016) and *AtMATE* from Arabidopsis (*AT3G21690*; percentage identity of 72.80%) (Liu *et al*., 2009; Koh *et al*., 2010), characterized as vacuolar membrane transporters (Appendix S2; Figure 3a). The *Manes.16G007900* and *Manes.16G00800* predicted topology of 12 transmembrane helices supports the annotation (Figure S5a,b) previously reported for Arabidopsis (Li *et al*., 2002), sorghum (Darbani *et al*., 2016) and blueberry (Chen *et al*., 2015) (Methods S5). The maximum‐likelihood tree using protein sequences from 241 HapMap individuals displayed a distinct clade distribution of 64 homozygous individuals for the SNP S16_773999 G:G allele (high HCN), colored red, and 114 homozygotes for the SNP S16_773999 A:A allele (low HCN), colored green. *Manihot esculenta* subsp*. flabellifolia* individuals (homozygote G:G) and the other wild accessions of *Manihot glaziovii* and *Manihot pruinosa* (homozygote A:A) were clustered in distinct clades (Figure 3b).

The stability of a protein to denaturation is calculated by measuring changes in free energy, and the higher and more positive the change in free energy is, the more stable the protein is against denaturation (Quan *et al*., 2016). We mined 36 single point mutation predictions in GBS and whole‐genome resequencing data (Ramu *et al*., 2017) for Manes.16G007900 and Manes.16G008000 proteins. In the observed 36 single point mutations across the two proteins, this value ranges from 0.26 to −4.00, with an average of −1.57 (Figure S5c(1–4); Methods S6; Table S10). The deleterious point mutations showed higher negative values in their structural change prediction. Mutations with sensitive stability changes can affect the motion and fluctuation of the target residues. All 36 point mutations, except one (Figure 2b, middle panel), had a negative change in free energy, indicating a loss of stability, conferring potential detrimental effects in protein function (Methods S6).

### Sweet and bitter cassava geographical distribution

**Figure 4:**
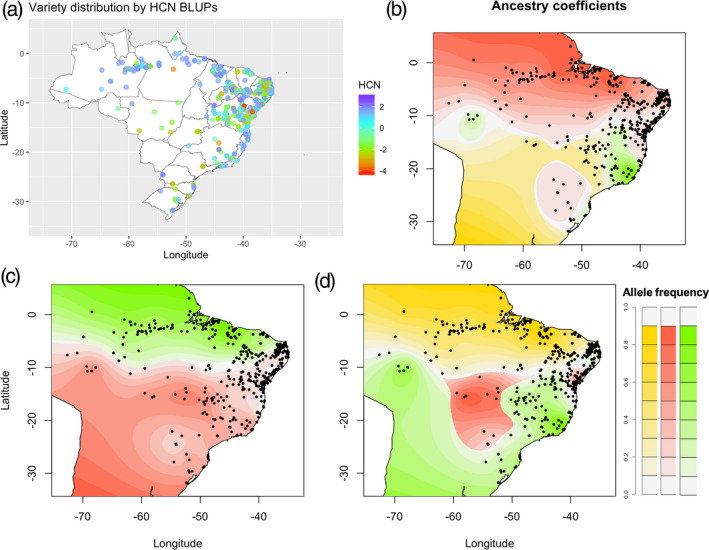
Spatial distribution of ancestral coefficients for HCN candidate single‐nucleotide polymorphisms (SNPs) using 1657 accessions. (a) Distribution of germplasm based on best linear unbiased prediction (BLUP) of HCN. Accessions with high HCN‐contributing alleles are grouped around the Amazonas and accessions with low HCN‐contributing alleles are grouped in other areas of Brazil. (b) Spatial distribution of allele frequency for HCN candidate loci in chromosome 14. (c) Spatial distribution of allele frequency for HCN candidate loci in chromosome 16. (d) Interactions of HCN candidate loci in chromosomes 14 and 16.

We represented the geographical distribution and HCN content of Brazilian germplasm recently characterized by Ogbonna *et al*. (2020) and presented a contrasted distribution (Figure 4a). Accessions with high HCN‐contributing alleles are grouped mostly around the Amazonas and low HCN‐contributing alleles are grouped in other areas of Brazil. Specifically, individuals with high HCN levels are mostly found around the Amazonian rivers and the coastal areas, whereas more variation in HCN content was observed in other regions of Brazil. The ancestry coefficient distribution for S16_773999, S14_5775892 and the joint haplotypes S16_773999 and S14_5775892 revealed three different ancestry coefficients for the candidate SNP S14_5775892 (Figure 4b), following an additive response (Figure 2c). Two different ancestry coefficients were observed for the candidate SNP S16_773999 (Figure 4c), following the complete dominant response observed (Figure 2d). The pseudohaplotype of candidate SNPs in chromosomes 14 and 16 shows the distribution of three ancestry coefficients (Figure 4d), indicating low, intermediate and high HCN ancestry coefficients (Appendix S3; Methods S7).

Using open‐source data (https://cassavabase.org; Methods S8), we explored the distribution of HCN across sub‐Saharan African data sets, including individuals assayed from 26 countries (Figure S6a; Table S11) and field trials carried out in different locations across Nigeria. This analysis indicated that, on average, Central and Southern Africa showed higher‐HCN varieties compared with West Africa (Figure S6b), whereas a trend towards lower HCN contents was detected in landraces compared with improved varieties (Figure S6c).

### Validating GWAS results in African and joint African and Latin American populations

**Figure 5:**
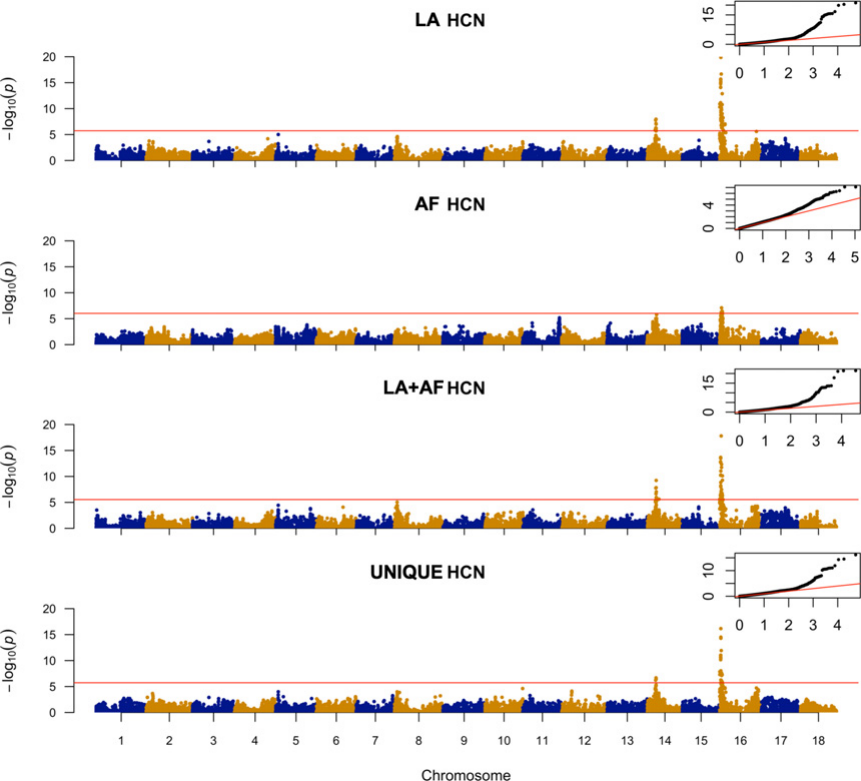
Manhattan plot from a mixed linear model (MLM‐LOCO) with the chromosome on which the candidate SNP is located excluded from calculating the genetic relationship matrix (GRM). The MLM‐LOCO summarizes the genome‐wide association results for HCN in Latin American (LA, Brazilian), African (AF), joint Latin American + African (LA + AF) and unique Latin American (UNIQUE LA) germplasms. Bonferroni significance threshold is shown in red. A quantile–quantile plot is inserted to demonstrate the observed and expected −log_10_ *P* for HCN.

Phenotypic distribution and variation for HCN content was measured in an African population of 636 individuals using the picrate titration method. HCN concentration varies from 1 to 9, with an average of 5.1 in the African population (Table S12). The *H* ^2^and *h* ^2^ values for HCN content were 0.27 and 0.26, respectively, which is less than that observed in Brazilian germplasm (Table S2). Genotype variance (*V*_g_) was higher than genotype‐by‐environment variance (*G*_g × e_), with the ratio (*V*_g × e_/*V*_g_) showing a high interaction of 0.86. The estimated deregressed BLUPs ranged from 0.0009 to 2.5638, with an average of 0.5242 (Table S13). After Bonferroni correction, with a −log_10_(0.05/53547) threshold of 6.029765, two significant peaks were identified on chromosomes 14 and 16, respectively (Figure 5, AF HCN; Table S14). A third peak was observed in chromosome 11 but did not cross the threshold for significance. The GWAS data set for HCN in African accessions showed peaks on chromosomes 14 and 16, with SNP S14_6612442 and SNP S16_1298874 showing the highest P values, congruent with the Brazilian GWAS data set.

For the African and Latin American combined analysis, phenotypic variation ranged between 1 and 9, with an average of 5.2 (Table S15). The *H* ^2^ and *h* ^2^ values for HCN content in African and Brazilian combined analysis were 0.50 and 0.38, respectively. The genotype variance (*V*_g_) was higher than genotype‐by‐environment variance (*G*_g × e_), with the *V*_g × e_/*V*_g_ ratio showing a lower interaction of 0.42, compared with that of the African population alone (Table S2). The estimated deregressed BLUPs (for the 1875 individuals used in GWAS) ranged from 0.0027 to 4.2266, with an average of 1.2545 (Table S16). After Bonferroni correction, two significant peaks were identified on chromosomes 14 and 16, respectively, corresponding to the earlier reported candidate SNPs (Figure 5, LA + AF HCN; Table S17). A whole‐genome imputation of the African–Brazilian data set using the HapMap as a reference panel for chromosome 16 (Figure S7a) further validates *Manes.16G007900* and the associated SNP S16_773999, based on an optimal *P* value (4.74E–22) (Methods S8; Table S18). Also see the distributions of phenotypes and deregressed BLUPs (Figure S8).

**Figure 6:**
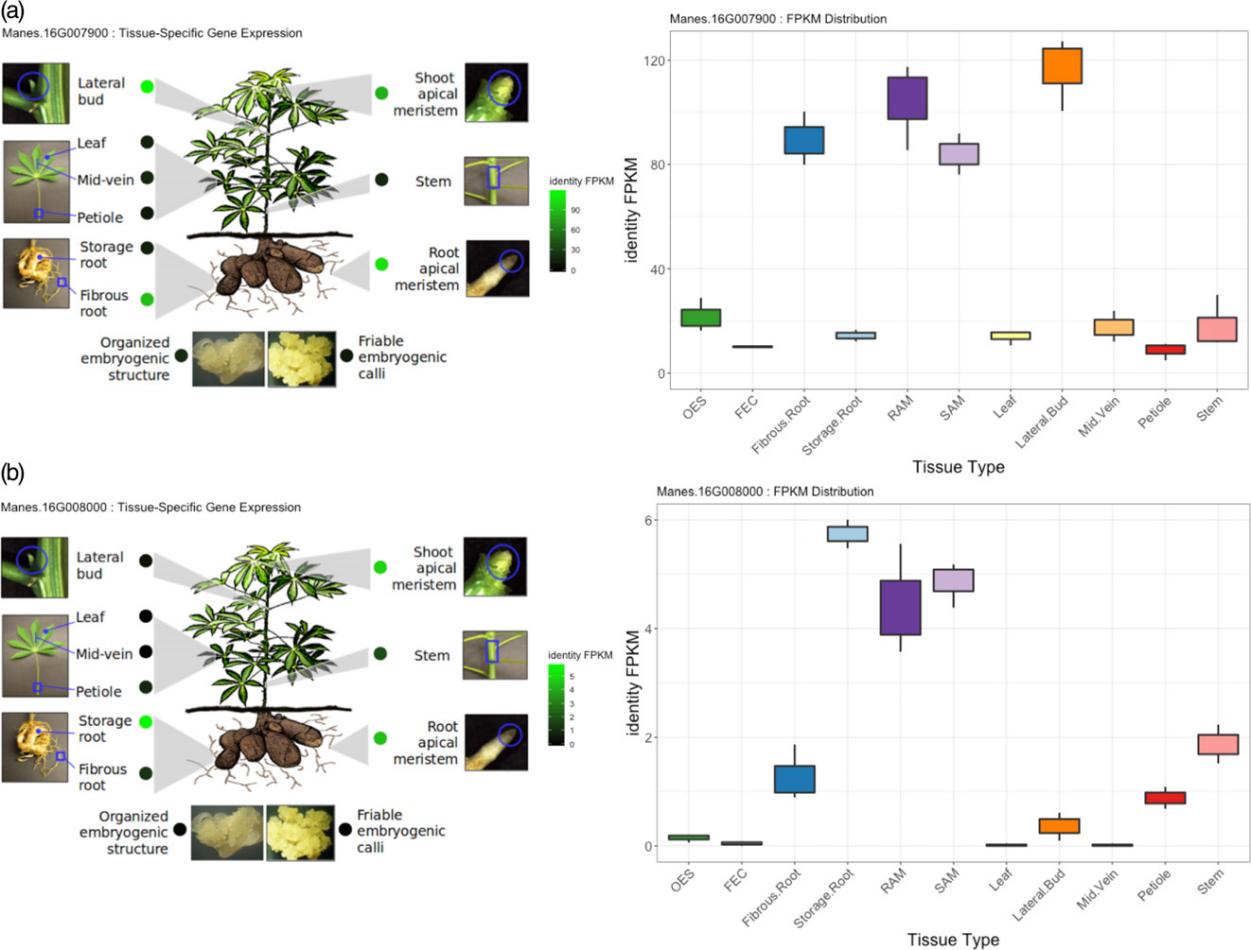
*Manes.16G007900* (a) and *Manes.16G008000* (b) tissues/organs expression profiles (FPKM, fragments per kilobase million) 3 months after planting African cassava accession TMEB204 (*Manihot esculenta*), sampled for gene expression (Wilson *et al*., 2017). TMEB204, an African variety, was assayed for HCN in a 1997 field experiment carried out at IITA Mokwa location (Nigeria), and forms part of the African data set, with average HCN content of 5.67 (min. = 5, max. = 7). TMEB204 allelic profile for candidate SNP S16_773999 on chromosome 16 is heterozygous, indicating the dominance of *Manes.16G007900* high‐HCN alleles.

We requested the available open‐source RNA‐sequencing data set on the molecular identities for 11 cassava tissue/organ types using the TMEB204 (TME204) cassava variety to evaluate gene expression (Wilson *et al*., 2017). Both *Manes.16G007900* and *Manes.16G008000* showed differential expression between storage and fibrous root, with *P* values of 5.00E–05 and 0.00065, respectively (Figure 6a,b). *Manes.16G007900* is differentially expressed between fibrous root and leaf, with FPKM (fragments per kilobase million) values of 13.9219 and 89.5362, respectively, whereas *Manes.16G008000* is not and shows low expression levels (Figure 6a,b). Selective sweep detection using HapMap WGS between cassava progenitors and Latin American and African accessions do not show sweeps overlapping with candidate and biosynthetic regions (Figures S9–S11).

## DISCUSSION

The potential of CG content in cassava varieties varies, even among the roots of the same plant (Gleadow and Møller, 2014). These variations are partly the result of genetics, environmental conditions and soil type (Bokanga *et al*., 1994; Jørgensen *et al*., 2005; Nzwalo and Cliff, 2011). Although germplasm from Latin America shows higher genetic variance and heritability (Brazil, *V*_g_ = 2.59, *H* ^2^ = 0.82, *h* ^2^ = 0.41; Colombia, *V*_g_ = 1.58, *H* ^2^ = 0.69), germplasm from African counterparts showed much less genetic variance and heritability (*V*_g_ = 0.21, *H* ^2^ = 0.27, *h* ^2^ = 0.26). Latin America/Brazil is the primary center of domestication, from where cassava was only introduced to Africa in the 16th century, which could explain the probable genetic bottleneck and the observed difference between the two populations (Bredeson *et al*., 2016). In addition, sweet and bitter cassava landraces are differentiated in Latin America but not in Africa. This is attributed to post‐introduction hybridization between sweet and bitter cassava, and the inconsistent transfer of ethnobotanical knowledge of use‐category management to Africa (Bradbury *et al*., 2013). The mislabeling of germplasm in Africa (Yabe *et al*., 2018) may also have contributed to the observed difference. These differences were also observed with the distribution plots for the individuals assayed in our analysis for HCN in Latin American (bimodal distribution) and African (almost normally distributed) populations. The observed differences between broad‐ and narrow‐sense heritability estimates, attributed to missing heritability, could be explained by: local epistasis interactions involving a few major genes and resulting in the observed of HCN in chromosome 16 (Akdemir and Jannink, 2015); large numbers of rare variants omitted through imputation (Yang *et al*., 2015); and the use of only biallelic subsets of filtered SNPs, leaving behind multiallelic loci, which may have contributed to additional variance.

Previous studies on the genetic architecture of HCN found two QTLs linked to loci SSRY105 and SSRY242 explaining 7 and 20% of the genetic variation in an S_1_ population (Kizito *et al*., 2007). Blasting the sequences of the loci revealed location SSRY105 on chromosome 14 (57 582 253 bp) of the cassava genome version 6.1 (https://phytozome.jgi.doe.gov), which was congruent with the region found on chromosome 14 associated with HCN variation in our current data sets. Whankaew *et al*. (2011), found five QTLs (CN09R1, CN09R2, CN09L1, CN09L2 and CN08R1) across two environments and years, but without any consistent QTLs. Their corresponding locations on the cassava genome version 6.1 were chromosomes 12 (CN09R1), 9 (CN09L1), 4 (CN09R2) and 3 (CN09L2). The sequences of SSRY242 and CN08R1 QTLs could not be found to specify their locations in the genome. These studies could not provide comprehensive information on the genetic basis for root HCN variation in cassava, as: (i) HCN content is affected by the environment; (ii) populations with distinct genetic backgrounds were used; (iii) HCN was assayed at different stages of field trials; and (iv) low marker densities were used, limiting the resolution and QTL detection power.

To provide comprehensive genetic architecture of HCN in cassava, we performed a GWAS using multiyear trials conducted in Brazil in 2016–2019 on individuals assayed for root HCN using the picrate titration method. Two major regions associated with HCN variation were identified in our data set: a stronger region in chromosome 16 (within the MATE efflux transporter coding region) and another region in chromosome 14 (within an integral membrane HPP family protein and H^+^‐ATPase coding regions). The validation of the genetic architecture of HCN in an African population, the joint GWAS analysis between Africa and Latin America (Brazil), and the whole‐genome imputation of the African–Brazilian data set using HapMap as a reference for chromosome 16 confirms these results and shows that the genetic architecture of HCN is conserved, based on our data sets. Homozygous reference alleles at the loci identified showing lower HCN content are in agreement with the finding that acyanogenic plants are homozygous recessive at one of the loci (Gleadow and Møller, 2014); however, such a homozygous cassava variety has yet to be identified given that they are recessive and difficult to discover because of the polyploid make‐up of cassava (Fregene and Puonti‐Kaerlas, 2002; Jennings and Iglesias, 2002). HCN is maintained in cultivated cassava populations from Africa and Latin America via the selection of high‐ and low‐HCN phenotypes under different environmental and herbivore pressures, leading to balanced selection. This phenomenon has been reported previously for HCN in *Trifolium repens* (white clover; Corkhill, 1942), *Sorghum bicolor* (Hansen *et al*., 2003) and *Trifolium* spp. (Kakes, 1997). More recently, selective sweep results between cultivated and cassava progenitors suggested that selection during domestication decreased CG content (Ramu *et al*., 2017).

Genome‐wide phylogenetic analysis of MATE genes in cassava, sorghum and Arabidopsis have suggested homology between our candidate gene and SbMATE2, a vacuolar membrane transporter characterized in sorghum for the CG dhurrin (Figure 3a). SbMATE2 functions in the accumulation of plant specialized metabolites such as flavonoids and alkaloids, and exports dhurrin and other hydroxynitrile glucosides, thereby providing protection against the self‐toxic biochemical nature of chemical defense compounds. The transport of the pH‐dependent unstable CG from its cytoplasmic site of production to the acidic vacuole is likely to contribute to reducing self‐toxicity (Darbani *et al*., 2016). Mechanistic studies on MATE transporters, such as the sorghum *SbMATE* gene, strongly suggest that its transport cycle could be driven by proton and/or cation (H^+^ or Na^+^) gradients (Doshi *et al*., 2017). SbMATE shows high affinity for Na^+^ and H^+^, and H^+^ constitutes the main electrochemical driving force in plants; hence, it is likely that H^+^ constitutes the main coupling ion for SbMATE. Darbani *et al*. (2016), reported that the biosynthetic gene cluster for dhurrin additionally includes a gene encoding a MATE transporter and glutathione *S*‐transferase gene for dhurrin uptake in *S. bicolor*.

Our study identified a MATE transporter on chromosome 16 and Na^+^ (from integral membrane HPP family protein) and a plasma membrane H^+^‐ATPase‐coupled transporter on chromosome 14, as involved in HCN content regulation. In cassava genome version 6.1, the HCN biosynthesis gene cluster is located on chromosome 12 within a 75‐kb interval, including a couple of changes in orientation and gene arrangement (Figure S3b). Interestingly, genome‐wide epistasis study did not reveal interactions with other parts of the genome, including the biosynthesis gene cluster region on chromosome 12. This finding contrasts with sorghum, where HCN biosynthesis and transport have been characterized within the same gene cluster (Darbani *et al*., 2016). This suggests a distinct evolutionary path for HCN regulation in cassava compared with sorghum. In view of this observation, we speculate that perhaps cassava domestication targeted the upstream or downstream genetic regulation steps of CG biosynthesis. In cassava, CGs are synthesized in the shoot apex (Andersen *et al*., 2000) and are then transported to the fibrous roots (Nartey, 1968; Koch *et al*., 1992; Jørgensen *et al*., 2005, Jørgensen *et al*., 2015). Jørgensen *et al*. (2005) reported a reduction of cyanogenic content in the leaves of RNAi transgenic cassava plants, but not in the roots, indicating a tissue‐specific regulation of HCN accumulation in roots. Candidate *Manes.16G007900* (chromosome 16) showed local epistasis interaction with a 1.36‐Mb region located 772 055–775 833 bp downstream. Epistatic effects that arise from alleles in gametic disequilibrium between closely located loci can contribute to long‐term responses, as recombination disrupts allelic combinations that have specific epistatic effects and the detection of epistasis is a key factor for explaining the missing heritability (Akdemir *et al*, 2017; Santantonio *et al*., 2019). This region spans over 54 biallelic ancestry‐informative single‐nucleotide markers fixed or nearly fixed between *M. esculenta* and *M. esculenta* subsp*. flabellifolia* (Ogbonna *et al*., 2020), suggesting that domestication can impact metabolic content targeting transport regulation (Wang *et al*., 2019), as earlier reported in maize and *Oryza sativa* (rice) (Sosso *et al*., 2015). In view of the above findings, we speculate that cassava domestication may have specifically targeted downstream genetic regulation steps of HCN biosynthesis. This is supported by the fact that root size (starch storage) and HCN content are the major traits of cassava domestication (Ramu *et al*., 2017). HCN is regulated in an oligogenic manner with two major loci explaining the variation across our data sets. To facilitate their use in breeding pipelines, SNPs tagging the major QTLs were converted to robust, high‐throughput, and easy to use competitive allele‐specific PCR (KASP) assays. The diagnostic markers for HCN (Table S7) are available for the global cassava improvement community through a commercial genotyping service provider under the High Throughput Genotyping Project (https://excellenceinbreeding.org/module3/kasp) via Intertek (https://www.intertek.com). We also observed that the closest homology observed for MATEs in cassava is in line with the results of the MATE protein alignment, which displays the highest homology between MATE genes on chromosome 16 and chromosome 17 (Figure 3a). This is congruent with previously identified paleotetraploidy in the cassava genome, where chromosomes 14 and 16 present partial conserved synteny with chromosomes 6 and 17, respectively (Bredeson *et al*., 2016). We found the candidate gene to be a paralog (68.59%) with *Manes.16G008000* and a homeolog (91.09%) with *Manes.17G038400*, indicating that our candidate had undergone double‐duplication events. This finding would need further investigation to clarify the potential fate of the observed tandem duplication (i.e. subfunctionalization or neofunctionalization). MATE candidate gene topology prediction suggests that our candidate MATE protein shares a similar topology in the membrane as those observed in the MATE protein family, and functions as an efflux carrier that mediates the extrusion of toxic substances (Brown *et al*., 1999; Morita *et al*., 2000; Li *et al*., 2002). Further functional characterization of the putative HCN transporters in cassava is required.

Allele mining and mutation prediction (Figure 2b) on the HapMap data set ensures that the current study captures the diversity of the HapMap panel. Moreover, DNA sequence analysis of *Manes.16G007900* across HapMap individuals shows that *M. esculenta* subsp*. flabellifolia* individuals are preferentially homozygous G:G (high‐HCN allele) for candidate SNP S16_773999, which is in line with its phenotypic characterization for HCN content by Perrut‐Lima *et al*. (2014). Interestingly, for the same candidate SNP, *M. glaziovii* and *M. pruinosa* gene sequences are all homozygous A:A (low‐HCN alleles) and cluster separately from *M. esculenta* subsp. *flabellifolia* (Figure 3b). However, sweeps on HapMap data groups (Latin American, African and progenitors) did not reveal selective sweeps associated with GWAS loci and biosynthesis clusters. Phenotypic spatial distribution analysis for sweet and bitter cassava in Brazil suggested that clinal variation occurred along subregion gradients, separating ancestral coefficients across ecoregions, and this agrees with the candidate marker response in the region regulating HCN variation in cassava. This reflects the role that environmental conditions and herbivore pressure played on HCN regulation and its synergy in maintaining balanced selection of HCN traits in cassava (Appendix S3).

In conclusion, we deciphered the genetic architecture of HCN in cassava and mapped the genetic region in chromosomes 16 and 14. The GWAS peak in chromosome 16 is strongly associated with the coding region of a MATE efflux protein, a CG transporter characterized in sorghum. In addition, the peaks on chromosome 14 are associated with the coding region of an integral membrane HPP family protein involved in nitrite transport activity and a plasma membrane H^+^‐ATPase‐mediated H^+^ influx, which potentially worked with MATE to participate in an HCN glucoside cotransport system.

The haplotype defined from the region in chromosomes 16 and 14 explained 36 and 8% of the total variance explained by the markers, whereas loci associated with the optimal P values explained 30 and 7% variance, respectively. The selected individuals carrying alleles for high‐ and low‐HCN in chromosomes 16 and 14 were further validated by designing KASP markers for breeding applications. This approach also found the same regions explaining the variance in an African data set for HCN, a joint data set for African and Latin American germplasm and a whole‐genome imputation of the African–Brazilian data set for chromosome 16, validating the candidate SNP. Sweet and bitter cassava have maintained their pre‐conquest distribution in Brazil, with breeding activities around northern and central regions creating a more balanced population with low, intermediate and high HCN clones.

The broader impact of this study was to understand the genetic mechanism of HCN content (total CGs) regulation in cassava root and the identification of closely linked SNP markers to enhance efficiency and cost‐effectiveness through marker‐assisted selection. Further steps can include: (i) the deployment of diagnostic markers for breeding applications; (ii) the development of co‐expression studies to further assess the source–sink relationship of HCN metabolism in multi‐environmental conditions and the impact of low HCN levels on pest and disease control in cassava; (iii) the breeding and introduction of low‐HCN cassava varieties that are high yielding and disease resistant to regions often affected by agricultural and health‐related crises, such as konzo, especially in sub‐Saharan Africa. Altogether, the present study consolidates our understanding of the genetic control of CG variation in cassava root and provides further insights into using genomics of diverse genetic background populations.

## EXPERIMENTAL PROCEDURES

### Plant material

A first data set including a total of 1389 accessions from the Cassava Germplasm Banks (CGB) of Brazilian Agricultural Research Corporation (Embrapa, https://www.embrapa.br), located in Cruz das Almas, Bahia, Brazil, were used for this study (Figure 1b). The region is tropical, with an average annual temperature of 24.5°C, a relative humidity of 80% and an annual precipitation of 1250 mm. The germplasm was collected from different cassava growing regions and ecosystems of Brazil, and consisted of landraces and modern breeding lines (de Oliveira *et al*., 2014; de Albuquerque *et al*., 2018).

A second data set including 1363 African accessions was obtained from the open‐source cassava breeding database (https://cassavabase.org). This data set comprises plant material from the International Institute of Tropical Agriculture (IITA, https://www.iita.org).

### DNA extraction

DNA extraction was performed following the protocol described by de Albuquerque *et al*. (2018) and Ogbonna *et al*. (2020) on the Embrapa CGB collection. Briefly, DNA was extracted from young leaves according to the cetyltrimethylammonium bromide (CTAB) protocol, as described by Doyle and Doyle (1987). The DNA was diluted in TE buffer (10 mm Tris‐HCl and 1 mm EDTA) to a final concentration of 60 ng μl^−1^, and the quality was checked by the digestion of 250 ng of genomic DNA from 10 random samples with the restriction enzyme *Eco*RI (New England Biolabs, https://www.neb.com).

### Genotyping

Genotyping, imputation, filtering methods and parameters were performed and determined as described in Ogbonna *et al*. (2020). Briefly, genotyping by sequencing (Elshire *et al*., 2011) was conducted using the *Ape*KI restriction enzyme (Rabbi *et al*., 2014) and Illumina sequencing read lengths of 150 bp. Marker genotypes were called with the TASSEL GBS pipeline V5 (Glaubitz *et al*., 2014) using cassava reference genome version 6.1, available from Phytozome (https://phytozome.jgi.doe.gov/pz/portal.html). After filtering (mean depth values, >5; missing data, <0.2; minor allele frequency, <0.01 per loci) and imputation (AR^2^ > 0.8) (Browning and Browning, 2009), the remaining markers were retained for downstream analysis.

### Phenotyping

#### Brazilian data set

Phenotypic data were collected on 1389 accessions over four trials in a single location with three replications each in 2016, 2017, 2018 and 2019. A total of 1246 accessions had both phenotypic and genotypic information and were retained for further analysis. HCN content, representing cassava root total CGs, was measured using the picrate titration method (Bradbury *et al*., 1999), as described by Fukuda *et al*. (2010). Briefly, this involves a qualitative determination of HCN potential in cassava root, and given that HCN potential varies considerably in plants, we assayed five or six plants in a plot and three roots per plant. A cross‐sectional sample (1 cm^3^) is taken at mid‐root for each root, between the peel and the center of the parenchyma. The cut root cube and five drops of toluene (methylbenzene or phenyl methane) are added to a glass test tube and the tube is tightly sealed with a stopper. To determine the qualitative score of HCN potential on a color scale of 1–9, a strip of Whatman filter paper is dipped into a freshly prepared alkaline picrate mixture until saturation. The saturated filter paper is then placed above the cut root cube in the glass tube and tightly sealed for 10–12 h before recording the color intensity (Maziya‐Dixon *et al*., 2007). For an HCN assay for Brazilian germplasm across 4 years, see Table S1.

#### African and Colombian data sets

African phenotypic data were collected from the breeding database Cassavabase (https://cassavabase.org), and included 18 locations, 23 years and 393 trials, for a total of 8244 accessions and a total of 33 523 observations from IITA (Figure S8c). Colombian phenotypic data included 41 locations, 11 years and 155 trials, for a total of 13 111 observations from the Centro Internacional de Agricultura Tropical (CIAT, https://ciat.cgiar.org). The phenotyping protocol was performed using the same protocol as for the Brazilian data set. A total of 636 unique accessions with phenotypic and genotypic information from 228 trials were retained for further analysis for the African data set.

### Statistical analyses

Trials across years were combined and BLUPs were estimated for each clone from 9138 observations on 1389 genotypes for HCN. We used the lme4 (Bates *et al*., 2015) package in r 3.4.2 (R. Core Team, 2015) to fit a mixed linear model (MLM) following the method described by Wolfe *et al*. (2016): $Y_{\mathrm{ijkl}}=\mu +C_{i}+\beta_{j}+r_{k}+d_{l}+\in_{\mathrm{ijkl}}$, where $\in_{\mathrm{ijkl}}∼N\left(0,\sigma_{\in }^{2}\right)$ is the residual variance and is assumed to be randomly distributed, $Y_{\mathrm{ijkl}}$ represents the phenotypic observations, µ is the grand mean, ci is the random effects for clone with $c_{i}∼N\left(0,\sigma_{i}^{2}\right)$, β*_j_* is a random effect for clone nested in combination of year, *r_k_* is a random effect for combination of year and rep, assumed to be normally distributed with , and *d_l_* is a fixed effect for years. Variance components from our mixed model were used to compute the broad‐sense heritability according to the method described by Holland *et al*. (2010). Briefly,

$$H^{2}=\frac{\sigma_{c}^{2}}{\sigma_{c}^{2}+\frac{\sigma_{t}^{2}}{\bar{t}}+\frac{\sigma_{r}^{2}}{\bar{r}}+\frac{\sigma_{\in }^{2}}{\bar{p}}},$$

where $\sigma_{c}^{2}$ is the clone variance, $\sigma_{t}^{2}$ is the variance associated with the clone by year, $\sigma_{r}^{2}$ is the variance for years by replications and $\sigma_{p}^{2}$ is the variance arising from error. $\bar{t}$, $\bar{r}$ and $\bar{p}$ are the harmonic mean number of years, replications and plots in which the clone was observed, respectively. Given that the number of observations per clone varies across the four years of data (replication varies from 1 to 9, with an average of 6), the bias induced by pre‐correction and induced heterogeneous residual variance (de Los Campos *et al*., 2013), the estimated BLUPs (differentially shrunken to the mean) were deregressed using:

$$\text{deregressed BLUP}=\frac{\text{BLUP}}{1‐\frac{\text{PEV}}{\sigma_{i}^{2}}},$$

where PEV is the prediction error variance for each clone and $\sigma_{i}^{2}$ is the variance for the clonal component. Figure S8 shows the deregressed BLUP distribution that was further used in the GWAS.

### GWAS analysis

We carried out mixed‐model genome‐wide association mapping using gcta (Yang *et al*., 2011). Specifically, we used MLM‐based association analysis with the chromosome on which the candidate SNP is located excluded from calculating the genetic relationship matrix (GRM). The model is , where *y* is the deregressed BLUP estimate, *a* is the mean term, *b* is the additive effect (fixed effect) of the candidate SNP to be tested for an association, *x* is the SNP genotype indicator variable, $g^{‐}$ is the accumulated effect of all SNPs except those on the chromosome where the candidate SNP is located and *e* is the residual. We used a Manhattan plot with Bonferroni threshold as a test of significant SNP associations, and compared the observed −log_10_(*P*) values against the expected values using the quantile–quantile plot. Local LD analysis was performed on significant regions in GWAS based on an *r* ^2^ threshold of >0.8 to identify candidate genes. GWAS was also performed on a unique set of 1536 individuals (GU panel) from (Ogbonna *et al*., 2020). This unique set was selected based on duplicate (identity–state) analysis on the total population of 3354 individuals to ensure efficient germplasm and resource management at the Brazilian cassava program, and to balance individual genetic contribution to population structure definition (Ogbonna *et al*., 2020).

### Candidate gene analysis

To investigate GWAS candidate regions further, we used the genomic resource from the cassava HapMap data (Ramu *et al*., 2017) to perform allele mining and predict genome‐wide allelic mutation effect using snpeff (Cingolani *et al*., 2012) and sift (Kumar *et al*., 2009).

### Phylogenetic analysis of candidate gene sequence

We obtained MATE whole‐genome protein sequences from *Arabidopsis thaliana* (v.10), *M. esculenta* (v.6.1) and *S. bicolor* (v3.11) genomes from Phytozome (https://phytozome.jgi.doe.gov/pz/portal.html). The sequences were submitted to the TransportTP prediction server (http://bioinfo3.noble.org/transporter/) for membrane domain identification and gene curation, according to the Transporter Classification Database (TCDB) guidelines (Saier *et al*., 2016). Sequences were aligned with clustal omega (Sievers *et al*., 2011) and a phylogenetic analysis was performed using a neighbor‐joining tree without distance corrections (Data S1 and S2). In addition, we generated the MATE candidate, *Manes.16G007900*, protein sequences from the cassava HapMap (Ramu *et al*., 2017). Briefly, *Manes16.G007900* annotated variants from HapMap II (ftp://ftp.cassavabase.org/HapMapII/) were used to generate coding sequences (CDSs) and translated protein sequences for 241 accessions in a fasta format. Subsequent alignment and maximum‐likelihood phylogenetic trees were generated using mafft (Katoh and Standley, 2013) and phyml (Guindon *et al*., 2010) through the NGphylogeny portal (https://ngphylogeny.fr/) (Lemoine *et al*., 2019).

### Open Research Badges

This article has earned an Open Data and Open Materials badges. Data and materials are available at ftp://ftp.cassavabase.org/manuscripts/Ogbonna_et_al_2020/ https://www.re3data.orgrepository/r3d100013440 and ftp://ftp.cassavabase.org/manuscripts/Ogbonna_et_al_2020/ https://www.re3data.orgrepository/r3d100013440

## Supporting information


**Figure S1.** Manhattan plot and LD plots for chromosomes 16 and 14.
**Figure S2.** Pearson correlation of the top‐five significant SNPs.
**Figure S3.** Schematic representation of the clustering of cyanogenic glucoside biosynthetic genes.
**Figure S4.** Differentiating loci between cultivated and cassava progenitors.
**Figure S5.** TMHMM posterior probability for transmembrane protein and mutation prediction.
**Figure S6.** Distribution of sweet and bitter cassava in Sub‐Saharan Africa.
**Figure S7.** Manhattan plot for whole‐genome imputed chromosomes 16.
**Figure S8.** Distribution of HCN assayed on Latin American and African cultivated accessions.
**Figure S9.** Selective sweeps between cassava progenitors and Latin American cultivated accessions.
**Figure S10.** Selective sweeps between Latin American and African cassava cultivated accessions.
**Figure S11.** Genetic (cM) vs. Physical (bp) positions.

**Table S1.** Raw HCN data set from Latin America (Embrapa, Brazil).
**Table S2.** Summary statistics, variance components and broad‐sense heritability for HCN.
**Table S3.** All 1389 BLUPs for the Latin American (Embrapa, Brazil) data set and the list of 1246 BLUPs with genotype information used for GWAS.
**Table S4.** Significant SNPs from Latin American data set (Embrapa, Brazil).
**Table S5.** Significant SNPs from GWAS on 523 unique individuals.
**Table S6.** Cultivated and cassava progenitor differentiating loci comparison: *Manihot* *esculenta* versus *Manihot* *esculenta* subsp*. flabellifolia*.
**Table S7.** Designed KASP marker sequences.
**Table S8.** HCN KASP segregation results.
**Table S9.** All 242 significant epistasis interaction pairs of SNPs higher than Bonferroni correction threshold (two‐way test result).
**Table S10.** Single point mutation prediction for *Manes.16G007900* and *Manes.16G008000*.
**Table S11.** List of countries and regions in sub‐Saharan Africa with their average BLUP values.
**Table S12.** Raw African data set phenotypes.
**Table S13.** African BLUPs used for GWAS analysis.
**Table S14.** Significant SNPs from African germplasm GWAS analysis.
**Table S15.** Raw African (IITA) and Latin American (Embrapa) phenotypes.
**Table S16.** All 1882 combined BLUPs for Africa (IITA) and Latin America (Embrapa) GWAS.
**Table S17.** Significant SNPs from African and Brazil germplasm.
**Table S18.** Significant SNPs from whole‐genome imputation of chromosome 16 GWAS using HapMap II and raw GBS data set; 5000 SNP windows were used.

**Data S1.** Whole‐genome sequence data set for all MATE genes in cassava, Arabidopsis and sorghum.
**Data S2.** Multiple sequence alignment for all MATE genes in cassava, Arabidopsis and sorghum.

**Methods S1.** Proportion of variance explained by markers.
**Methods S2.** Genome‐wide epistasis interactions.
**Methods S3.** Cultivated and cassava progenitor differentiating loci analysis.
**Methods S4.** KASPAR marker design and assessment.
**Methods S5.** Candidate gene protein topology and structure prediction.
**Methods S6.** Single point mutation prediction.
**Methods S7.** Geographical distribution of HCN.
**Methods S8.** GWAS in African population and joint African and Latin American analysis.

**Appendix S1**. Population structure analysis.
**Appendix S2**. Phylogenetic tree.
**Appendix S3**. Geographical distribution of sweet and bitter cassava.

## Data Availability

Genotyping (SNP) data used in this study were deposited on cassavabase.org hosted at ftp://ftp.cassavabase.org/manuscripts/Ogbonna_et_al_2020/gwas_manuscript.
