# Supplementary material for: Large‐scale genome‐wide association study, using historical data, identifies conserved genetic architecture of cyanogenic glucoside content in cassava (Manihot esculenta Crantz) root
Source: Plant J. 2020 Dec 18;105(3):754–70. doi: 10.1111/tpj.15071 (PMC7898387; doi:10.1111/tpj.15071)
Supplement: Supplementary file 1 — Figure S1. Manhattan plot and LD plots for chromosomes 16 and 14. Figure S2. Pearson correlation of the top‐five significant SNPs. Figure S3. Schematic representation of the clustering of cyanogenic glucoside biosynthetic genes. Figure S4. Differentiating loci between cultivated and cassava progenitors. Figure S5. TMHMM posterior probability for transmembrane protein and mutation prediction. Figure S6. Distribution of sweet and bitter cassava in Sub‐Saharan Africa. Figure S7. Manhattan plot for whole‐genome imputed chromosomes 16. Figure S8. Distribution of HCN assayed on Latin American and African cultivated accessions. Figure S9. Selective sweeps between cassava progenitors and Latin American cultivated accessions. Figure S10. Selective sweeps between Latin American and African cassava cultivated accessions. Figure S11. Genetic (cM) vs. Physical (bp) positions. [file TPJ-105-754-s001.pdf]

Title: **Large scale GWAS using historical data identifies a conserved genetic architecture of cyanogenic glucoside content in cassava *(Manihot esculenta Crantz.*) root**

Authors: Alex C Ogbonna[1,2], Luciano Rogerio Braatz de Andrade[3], Ismail Y. Rabbi[4], Lukas A. Mueller[1,2], Eder Jorge de Oliveira[3] and Guillaume J. Bauchet[2]

[1] Cornell University, Ithaca, NY, USA. [2] Boyce Thompson Institute for Plant Research, Ithaca, NY, USA. [3] Embrapa Mandioca e Fruticultura, Cruz das Almas, BA - Brazil.[4] International institute of Tropical Agriculture, Ibadan, Oyo state, Nigeria

The following **Supporting** Information is available for this article:

Supporting Figure S1

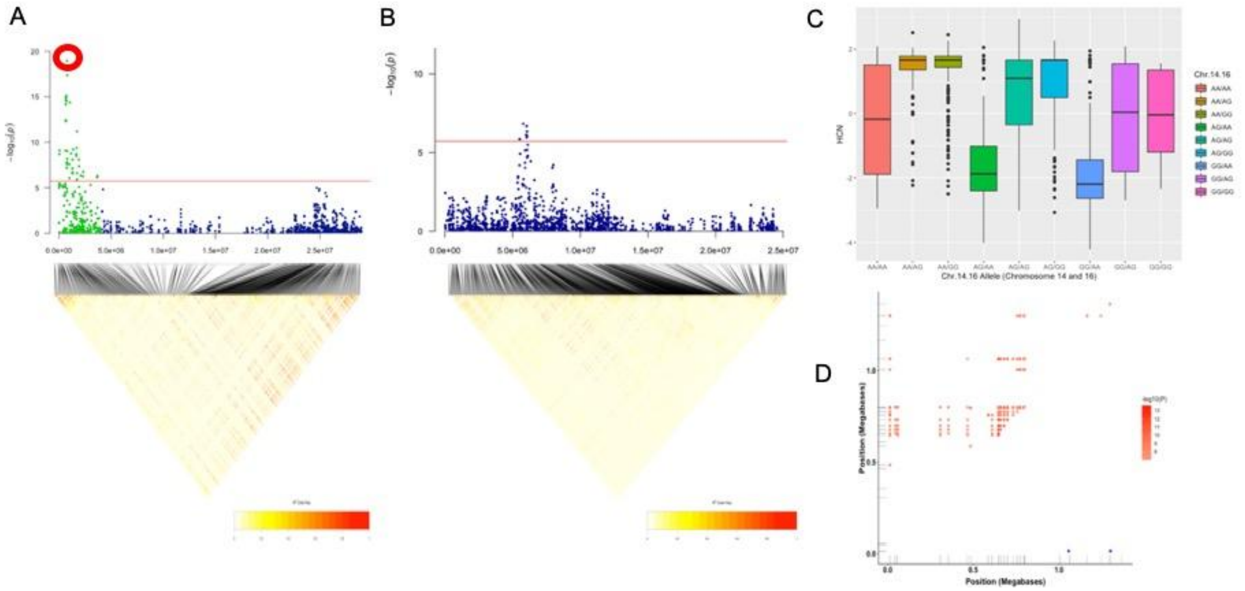

Manhattan plot from mixed linear model (MLM-LOCO) of chromosomes associated with cyanide variation in Latin American cassava. Below each Manhattan plot is a linkage disequilibrium (LD) heatmap for each chromosome showing pairwise squared correlation of alleles between markers. Bonferroni significance threshold is shown in red. **(A)** Manhattan plot of chromosome 16 showing candidate SNP for cyanide variation. The red circle indicates the candidate SNP. **(B)** Manhattan plot of chromosome 14 showing peak for cyanide variation. **(C)** Box plot showing the distribution of HCN for the combined effect (epistatic

interaction) of the top significant markers for chromosome 16 and 14. HCN BLUP values were plotted on the Y-axis, while allelic effects of the candidate SNPs from chromosomes 14 and 16 combined on the X-axis. **(D)** Epistasis Interactions for HCN variation in LA germplasm. The upper triangle of the plot (red) represents 242 significant epistasis interactions (Bonferroni correction threshold, 0.05/1131*(1131-1)/2) for chromosome 16. The lower triangle of the plot (blue) shows the 3 separated interactions, with 2 of them overlapping at 1.286336 Megabases. The Y and X-axis of the plot are the positions in Megabases of the set of SNP1 and SNP2 interacting markers, respectively.

Supporting Figure S2.

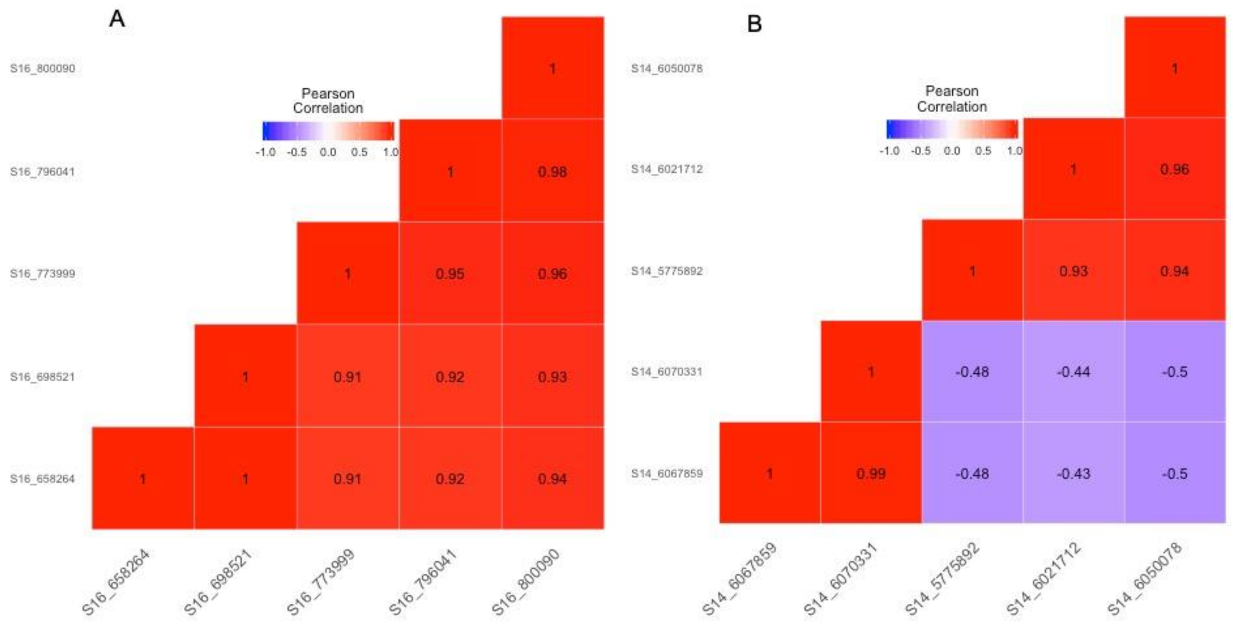

Pearson correlation using corrplot function in R package version 3.6.3 (2020-02-29) of top 5 significant SNPs in chromosome 16 (A) and 14 (B).

Supporting Figure S3.

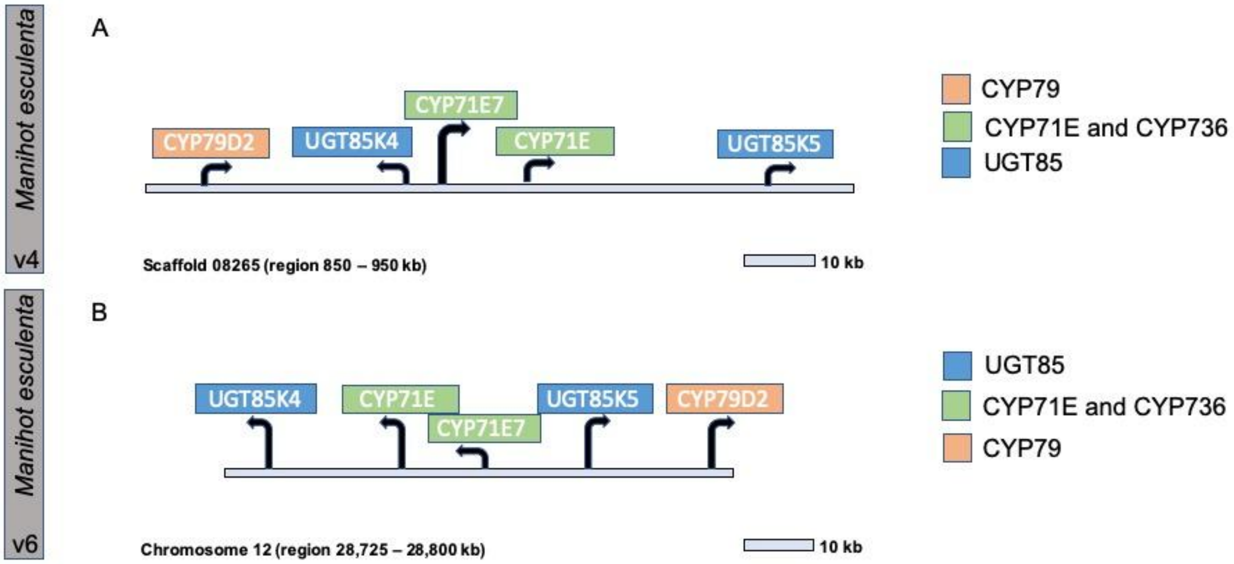

Schematic representation of the clustering of cyanogenic glucoside biosynthetic genes in the genome of *M. esculenta*. Functional genes are presented by arrows indicating their orientation. Confirmed genes in cyanogenic glucoside biosynthesis are labelled above each bar, with *CYP79* genes in pink, *CYP71E* and *CYP736* genes in green, and *UGT85* genes in blue. The sequences of the genes were retrieved and blasted against cassava genome version 6.1 on phytozome (http://phytozome.jgi.doe.gov). (A) Genome version Cassava4.1 (B) Genome draft version Cassava6.1. ***Adapted from Takos et al., the plant journal, 2011.***

Supporting Figure S4.

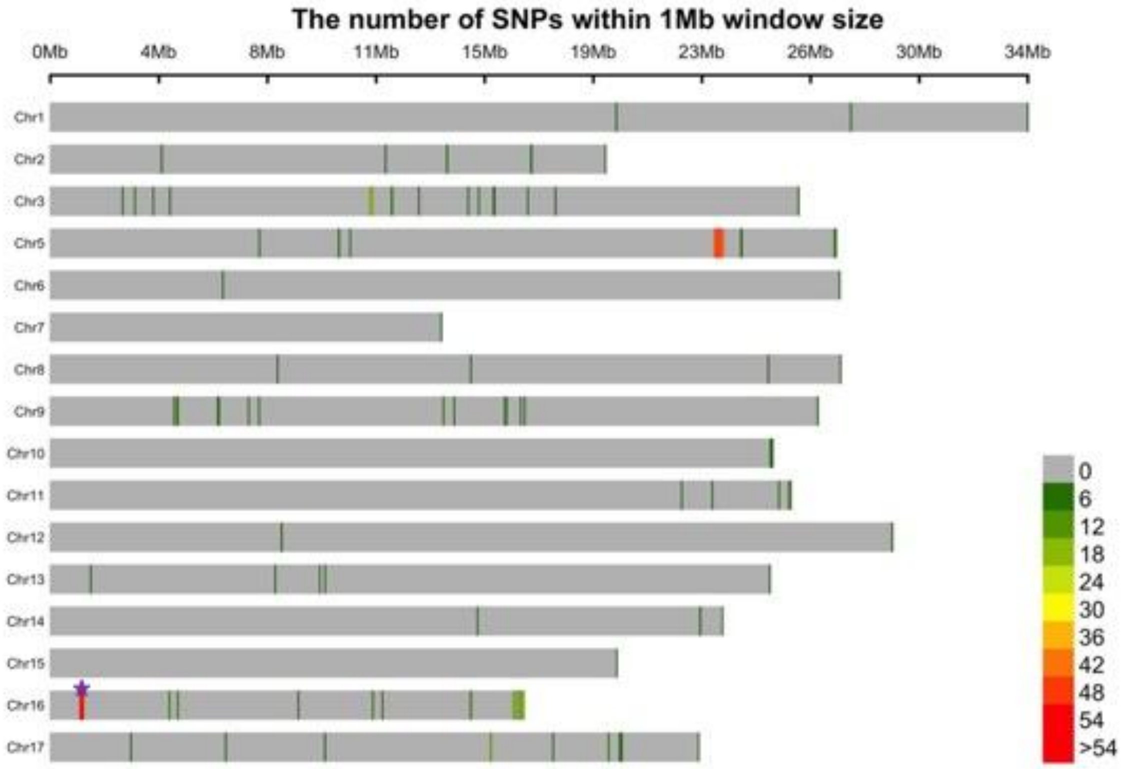

The plot shows the distribution of 294 biallelic ancestry-informative single-nucleotide markers that represent fixed, or nearly fixed, differences between *M. esculenta* and *M. flabellifolia in the hapmap II WGS dataset*. The legend scale indicates the number of SNPs within 1Mb window size. The purple star represents the identified region for cyanide regulation in cassava.

Supporting Figure S5.

A

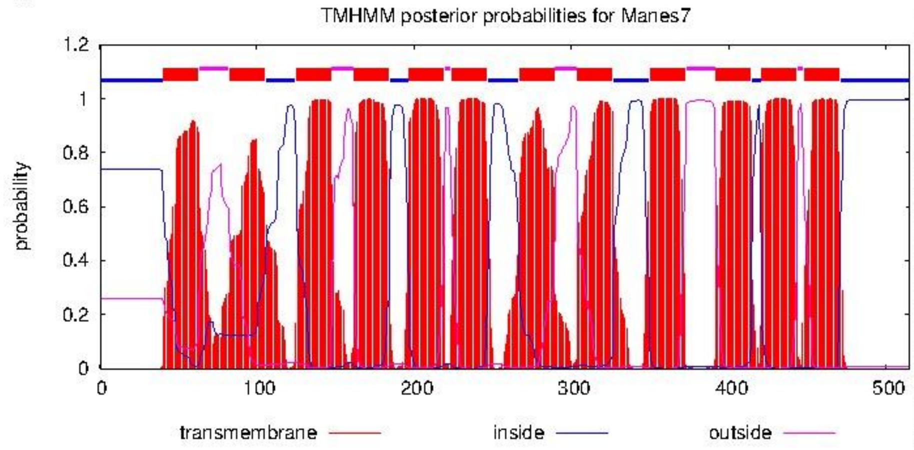

TMHMM result

```
# Manes7 Length: 515
# Manes7 Number of predicted TMHs:  12
# Manes7 Exp number of AAs in TMHs: 265.87223
# Manes7 Exp number, first 60 AAs:  13.97659
# Manes7 Total prob of N-in:        0.73944
# Manes7 POSSIBLE N-term signal sequence
Manes7  TMHMM2.0        inside        1     40
Manes7  TMHMM2.0        TMhelix      41     63
Manes7  TMHMM2.0        outside      64     82
Manes7  TMHMM2.0        TMhelix      83    105
Manes7  TMHMM2.0        inside      106    124
Manes7  TMHMM2.0        TMhelix     125    147
Manes7  TMHMM2.0        outside     148    161
Manes7  TMHMM2.0        TMhelix     162    184
Manes7  TMHMM2.0        inside      185    196
Manes7  TMHMM2.0        TMhelix     197    219
Manes7  TMHMM2.0        outside     220    223
Manes7  TMHMM2.0        TMhelix     224    246
Manes7  TMHMM2.0        inside      247    266
Manes7  TMHMM2.0        TMhelix     267    289
Manes7  TMHMM2.0        outside     290    303
Manes7  TMHMM2.0        TMhelix     304    326
Manes7  TMHMM2.0        inside      327    349
Manes7  TMHMM2.0        TMhelix     350    372
Manes7  TMHMM2.0        outside     373    391
Manes7  TMHMM2.0        TMhelix     392    414
Manes7  TMHMM2.0        inside      415    420
Manes7  TMHMM2.0        TMhelix     421    443
Manes7  TMHMM2.0        outside     444    447
Manes7  TMHMM2.0        TMhelix     448    470
Manes7  TMHMM2.0        inside      471    515
```

B

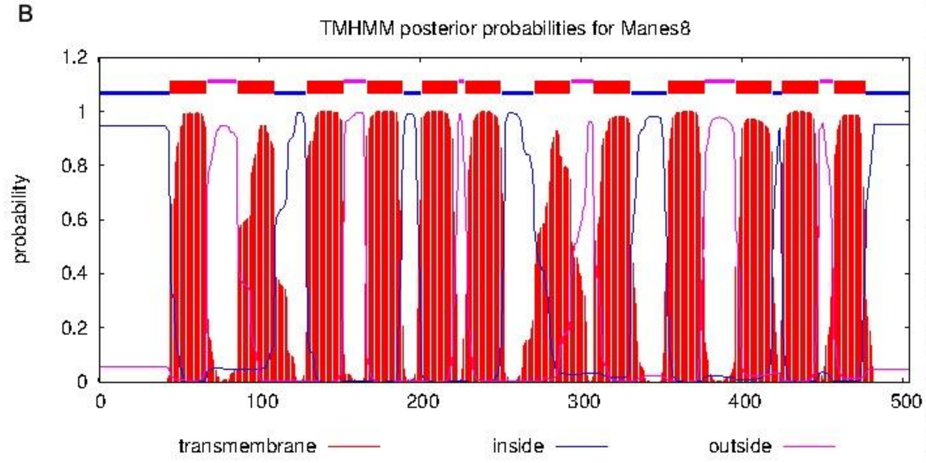

TMHMM result

```
# Manes8 Length: 504
# Manes8 Number of predicted TMHs:  12
# Manes8 Exp number of AAs in TMHs: 264.87498
# Manes8 Exp number, first 60 AAs:  14.37589
# Manes8 Total prob of N-in:        0.94595
# Manes8 POSSIBLE N-term signal sequence
Manes8  TMHMM2.0        inside        1     44
Manes8  TMHMM2.0        TMhelix      45     67
Manes8  TMHMM2.0        outside      68     86
Manes8  TMHMM2.0        TMhelix      87    109
Manes8  TMHMM2.0        inside      110    129
Manes8  TMHMM2.0        TMhelix     130    152
Manes8  TMHMM2.0        outside     153    166
Manes8  TMHMM2.0        TMhelix     167    189
Manes8  TMHMM2.0        inside      190    200
Manes8  TMHMM2.0        TMhelix     201    223
Manes8  TMHMM2.0        outside     224    227
Manes8  TMHMM2.0        TMhelix     228    250
Manes8  TMHMM2.0        inside      251    270
Manes8  TMHMM2.0        TMhelix     271    293
Manes8  TMHMM2.0        outside     294    307
Manes8  TMHMM2.0        TMhelix     308    330
Manes8  TMHMM2.0        inside      331    353
Manes8  TMHMM2.0        TMhelix     354    376
Manes8  TMHMM2.0        outside     377    395
Manes8  TMHMM2.0        TMhelix     396    418
Manes8  TMHMM2.0        inside      419    424
Manes8  TMHMM2.0        TMhelix     425    447
Manes8  TMHMM2.0        outside     448    456
Manes8  TMHMM2.0        TMhelix     457    476
Manes8  TMHMM2.0        inside      477    504
```

C

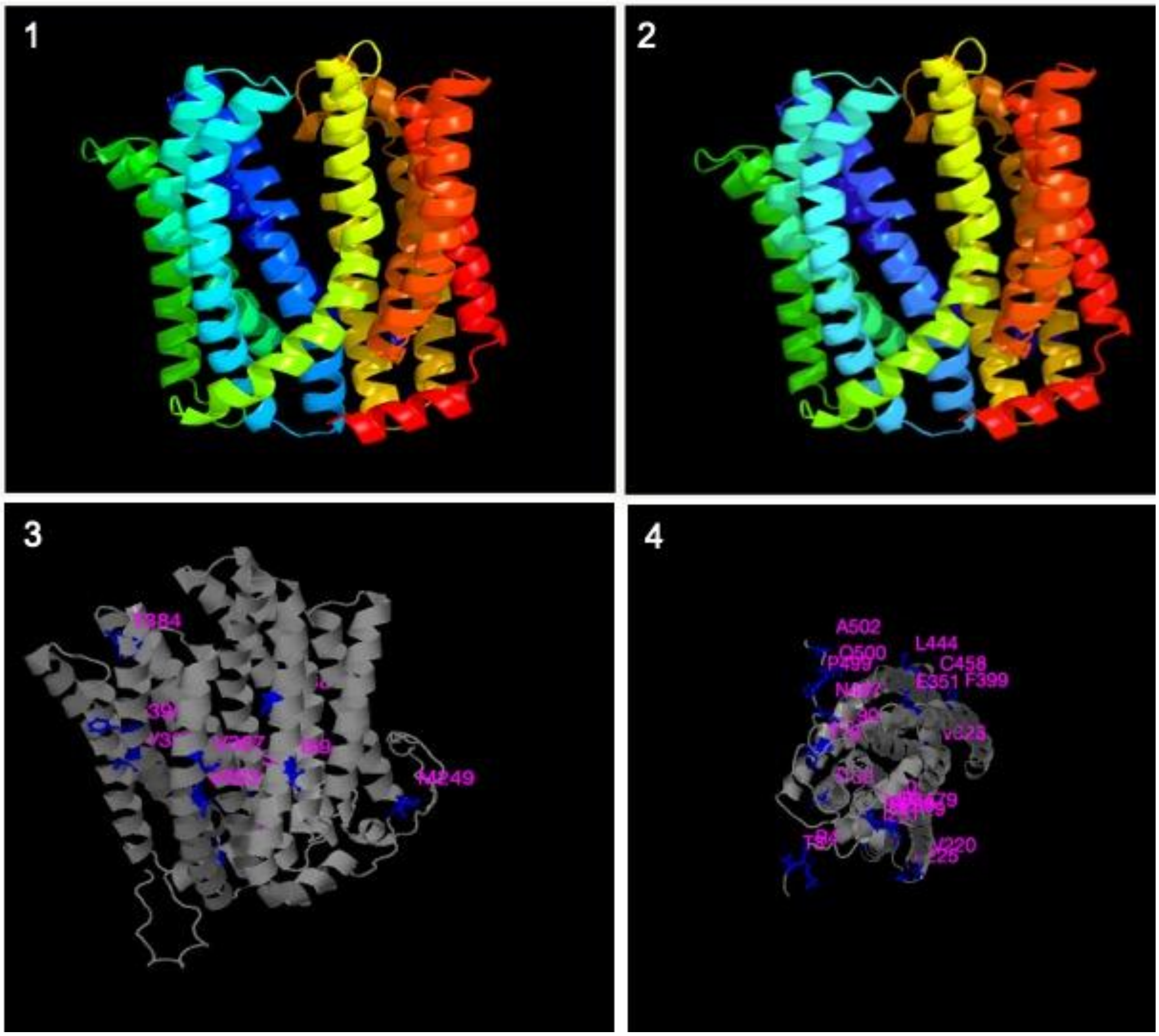

Transmembrane Helices prediction using Hidden Markov Model (TMHMM) posterior probability for transmembrane helix, inside, or outside displayed for the MATE transport family protein (Manes.16G007900.1 and Manes.16G008000.1) from cassava via (https://services.healthtech.dtu.dk/service.php?TMHMM-2.0). TMHMM is described in (Krogh et al., 2001), *Journal of Molecular Biology)*. **(A)** TMHMM results for Manes.16G007900.1 (here named Manes7), statistics and a list of the location of the predicted transmembrane helices and the predicted location of the intervening loop regions. Protein length: 515 amino acids, Number of predicted transmembrane (TM) helices: 12, Expected number of amino acids in TM helices: 265.87, Expected number of amino acids in TM helices in the first 60 amino acids of the protein: 13.98, Probability that the N-term is on the cytoplasmic side of the membrane: 0.74. The prediction gives the most probable location and orientation of transmembrane helices in the sequence. **(B)** TMHMM results for Manes.16G008000.1 protein (here named Manes8), statistics and a list of the location of the predicted transmembrane helices and the predicted location of the intervening loop regions. Protein length: 504, Number of predicted TM helices:

12, Expected number of amino acids in TM helices: 264.87, Expected number of amino acids in TM helices in the first 60 amino acids of the protein: 14.38, Probability that the N-term is on the cytoplasmic side of the membrane: 0.95. The prediction gives the most probable location and orientation of transmembrane helices in the sequence. **(C)** The protein structure was modelled using the Phyre2 server (http://www.sbg.bio.ic.ac.uk/phyre2) following the procedure outlined in Kelley *et al.* (2015). **(1)** Manes.16G007900: the model is based on the Crystal structure of eukaryotic MATE transporter AtDTX14(PDB ID: 5Y50A) with 100% confidence over 443 residues (36% of the sequence) (He et al. 2010). **(2)** Manes.16G008000**:** the model is based on the Crystal structure of eukaryotic MATE transporter AtDTX14(PDB ID: 5Y50A, (Miyauchi et al. 2017)) with 100% confidence over 446 residues (88% of the sequence) (He et al. 2010). **(3)** Single point mutation prediction positions for Manes.16G007900. **(4)** Single point mutation prediction positions for Manes.16G008000. STRUM (https://zhanglab.ccmb.med.umich.edu/STRUM/) was used to predict structural changes based on single point mutations.

Supporting Figure S6.

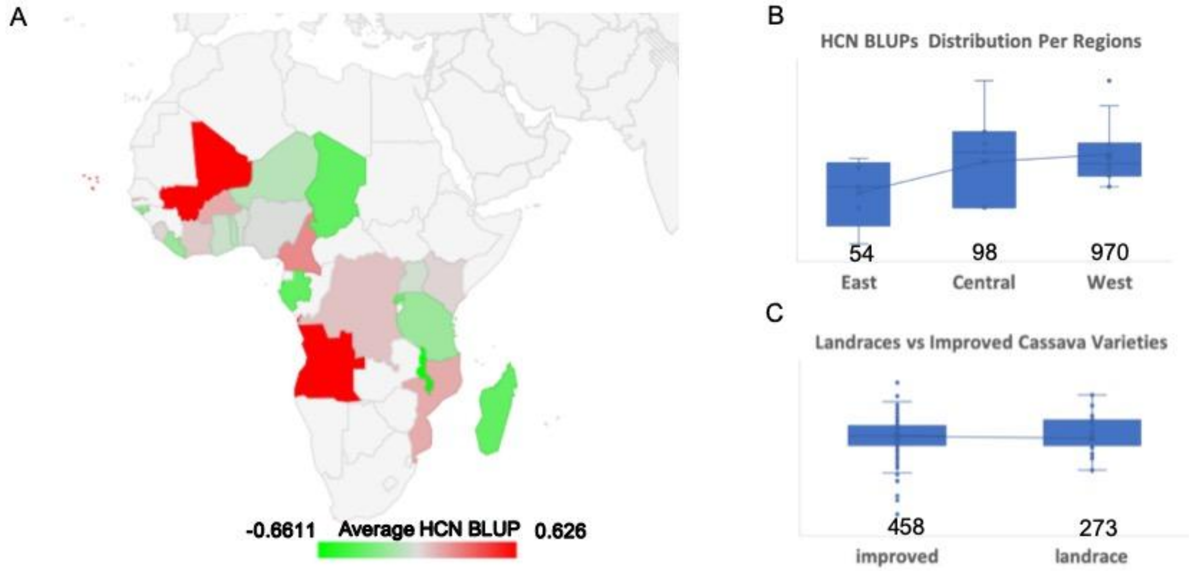

Cyanide (HCN) distribution by country and region for 1,156 accessions with country of origin in our African dataset (**Supporting Table S11**). **(A)** Distribution of accessions based on average best linear unbiased prediction (BLUP) of HCN across countries in sub-Saharan Africa. Average HCN is higher in Central Africa where Konzo disease has prevailed. **(B)** Distribution of accessions based on average best linear unbiased prediction (BLUP) of HCN across regions of Africa, including data for 54, 98 and 970 accessions for East, Central and West Africa respectively. **(C) L**andraces vs improved accessions comparison including 458 improved and 273 landrace accessions.

Supporting Figure S7.

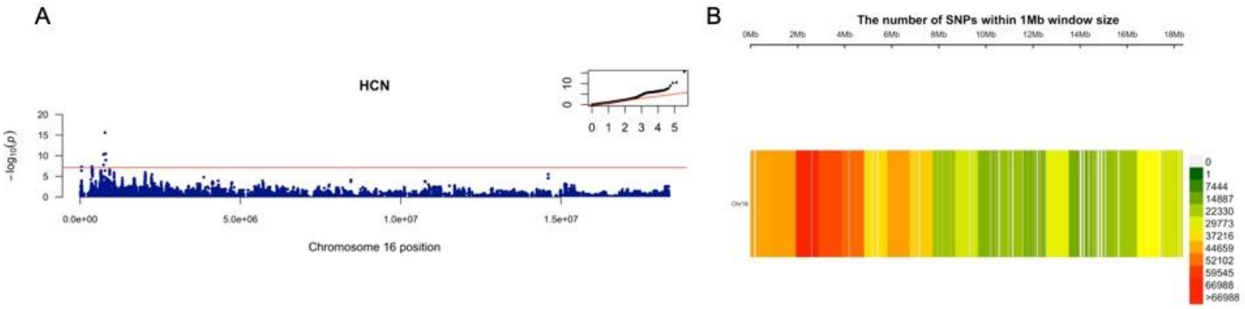

(A) Manhattan plot of Whole-Genome imputed chromosome 16 based on HapMapII (up to about 18 Megabases) from mixed linear model (MLM) using raw GBS from joint Latin American + African (LA+AF) germplasm. A total of 643,750 SNPs was imputed around 18 Megabases of chromosome 16 using 1877 individuals. The Bonferroni significance threshold is shown in red [7.109747, -log10(0.05/643750)]. A quantile-quantile plot is inserted to demonstrate the observed and expected -log10 of P-value for HCN. (B) Imputed single nucleotide polymorphism (SNP) density of joint Latin American + African (LA+AF) germplasm. The legend scale indicates the number of SNPs within 1Mb window size. The plot shows the distribution of SNPs across chromosome 16.

Supporting Figure S8.

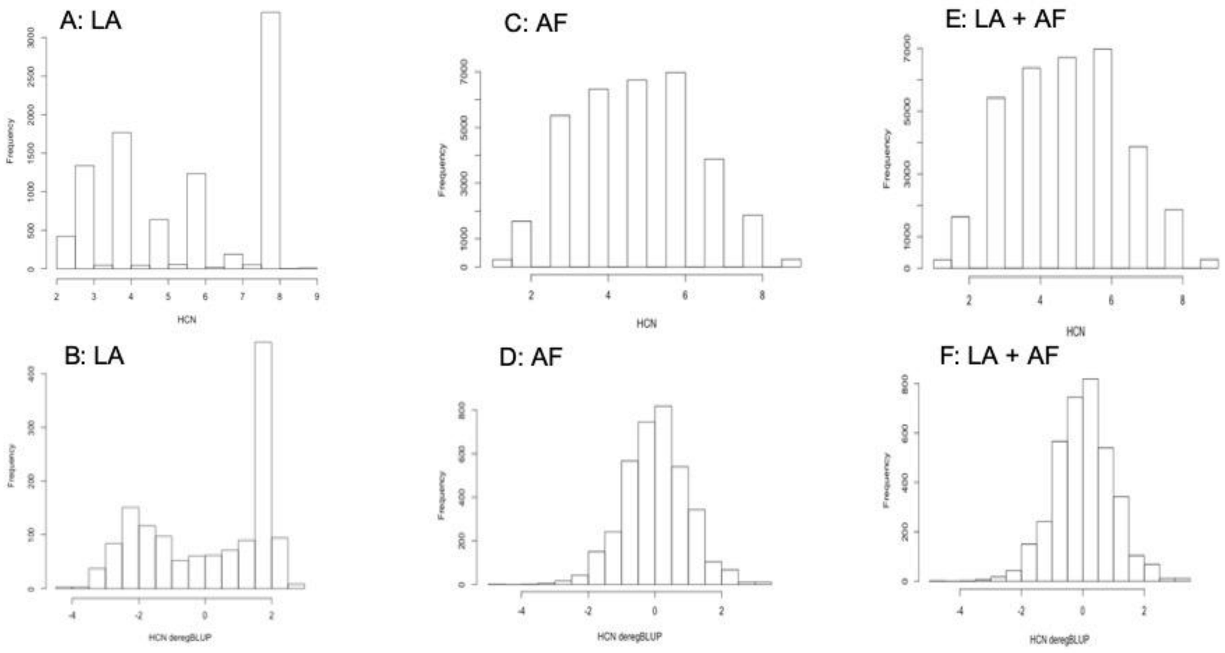

Distribution of HCN assayed for Latin American (LA, Brazilian), African (AF) and joint Latin American + African (LA+AF) germplasms. **(A)** Raw HCN phenotype scores for LA. **(B)** De-regressed HCN BLUPs for LA. **(C)** Raw HCN phenotype scores for AF. **(D)** De-regressed HCN BLUPs for AF. **(E)** Raw HCN phenotype scores for LA + AF. **(F)** De-regressed HCN BLUPs for LA + AF.

Supporting Figure S9.

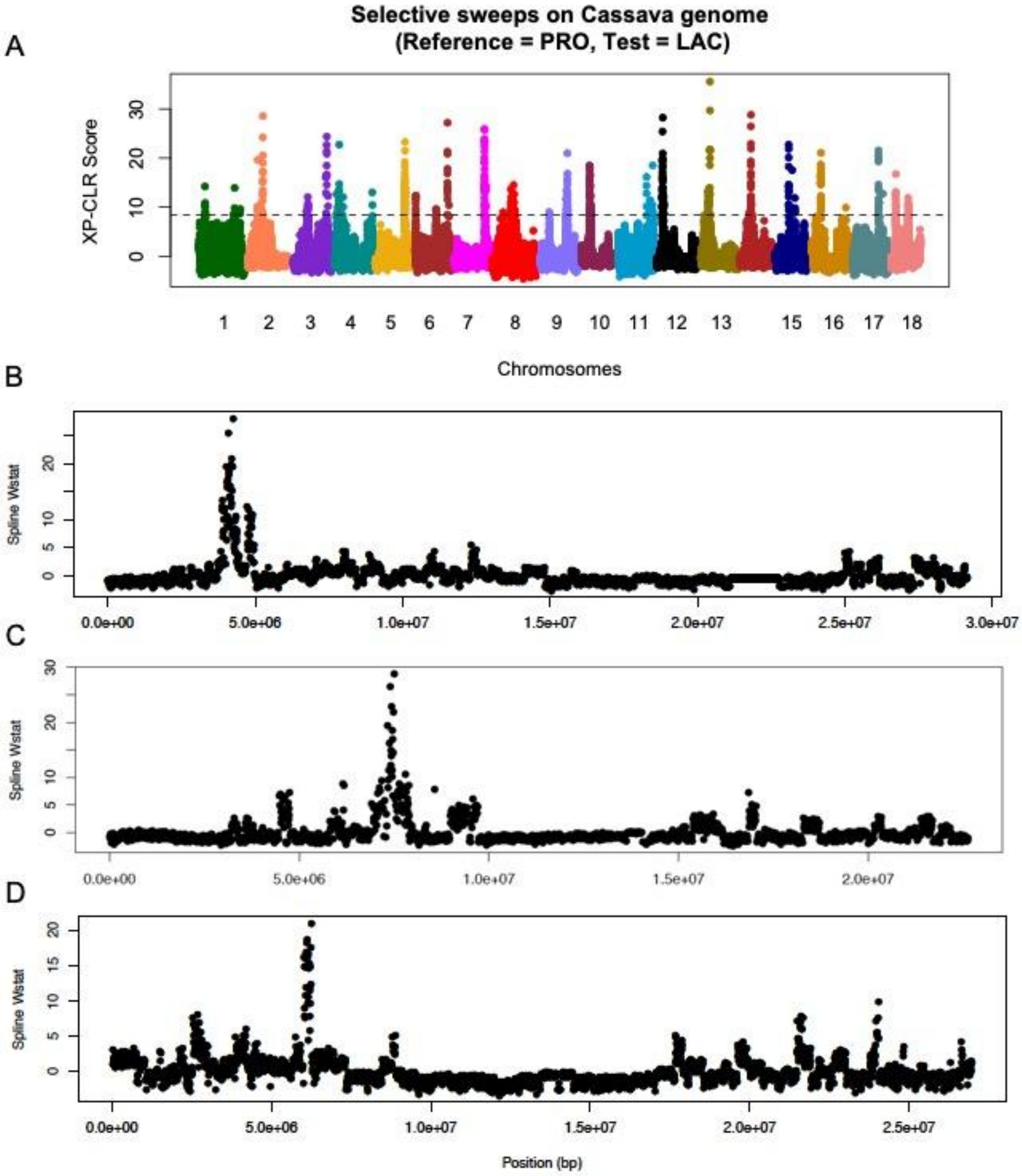

Selective sweep detection in Cassava HapMap II between Progenitors (PRO) and Latin American (LAC) accessions, following the approach described in Ramu et al (Ramu et al., 2017) (A) whole genome scan. (B) chromosome 12. (C) chromosome 14. (D) chromosome 16.

Supporting Figure S10.

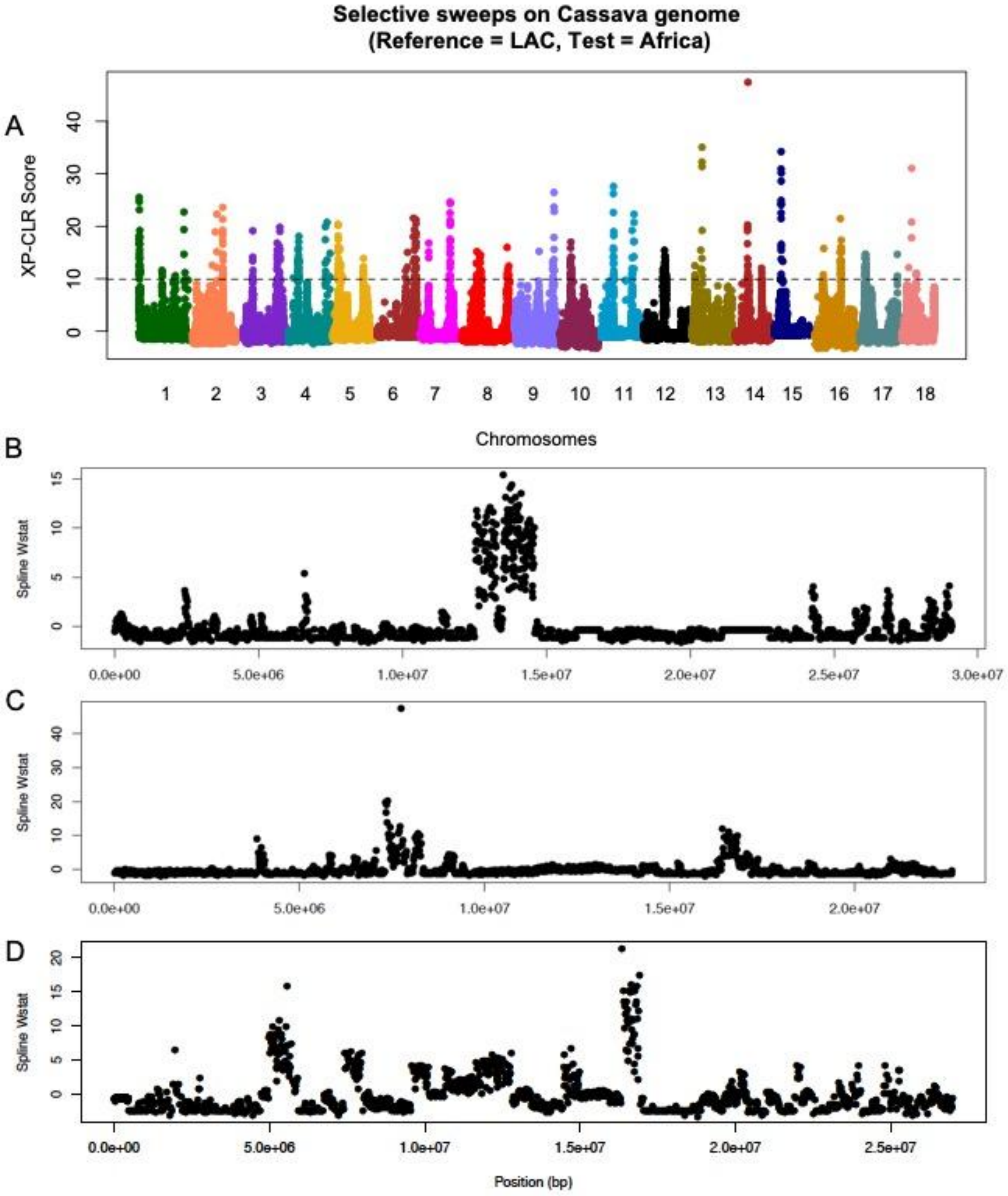

Selective sweep detection between Latin American (LAC) versus African accessions using the Cassava HapMap dataset following the approach described in Ramu et al (Ramu et al. 2017). (A) across the genome. (B) chromosome 12. (C) chromosome 14. (D) chromosome 16.

Supporting Figure S11.

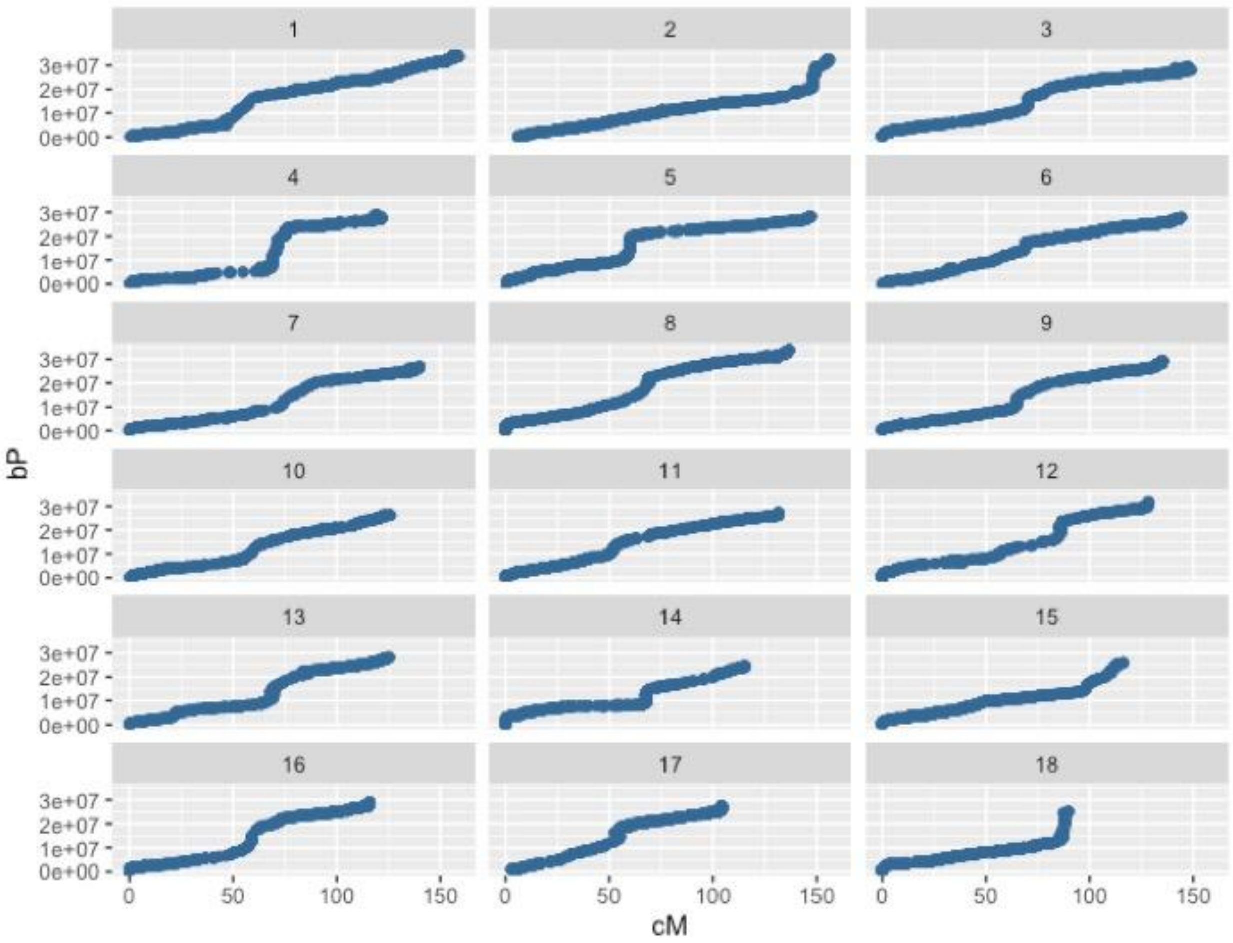

Projection of physical (bp) positions of Brazilian GBS marker set physical positions on updated International Cassava Genetic Map Consortium (ICGMC) linkage map based on method described in Wolfe et al (Wolfe et al. 2019) Scatterplots of genetic position vs. reference genome v6 physical positions for all 18 linkage groups for use in sweep detection analysis.

References

Kelley, L. A. *et al.* (2015) The Phyre2 web portal for protein modeling, prediction and analysis, *Nature protocols*, 10(6), pp. 845–858

Krogh, A. *et al.* (2001) Predicting transmembrane protein topology with a hidden Markov model: application to complete genomes, *Journal of molecular biology*, 305(3), pp. 567–580

Miyauchi, H. *et al.* (2017) Structural basis for xenobiotic extrusion by eukaryotic MATE transporter, *Nature communications*, 8(1), 1633

Quan, L., Lv, Q. and Zhang, Y. (2016) STRUM: structure-based prediction of protein stability changes upon single-point mutation, *Bioinformatics*, 2936–2946. doi: 10.1093/bioinformatics/btw361

Ramu, P. *et al.* (2017) Cassava haplotype map highlights fixation of deleterious mutations during clonal propagation, *Nature genetics*, 49(6), 959–963
