# Supplementary figures and images for: Large‐scale genome‐wide association study, using historical data, identifies conserved genetic architecture of cyanogenic glucoside content in cassava (Manihot esculenta Crantz) root
Source: Plant J. 2020 Dec 18;105(3):754–70. doi: 10.1111/tpj.15071 (PMC7898387; doi:10.1111/tpj.15071)

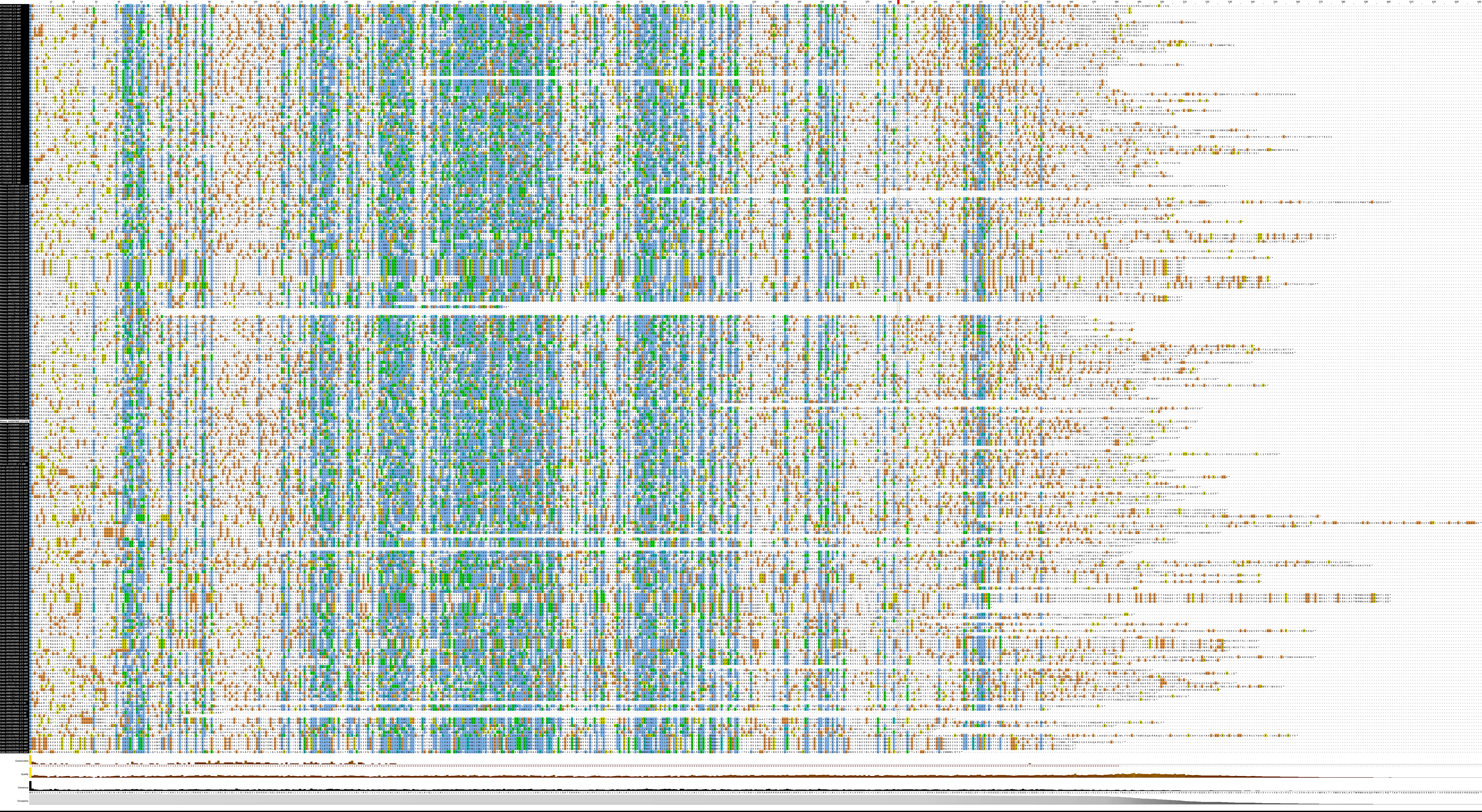

Supplement: Supplementary file 3 — Data S1. Whole‐genome sequence data set for all MATE genes in cassava, Arabidopsis and sorghum. Data S2. Multiple sequence alignment for all MATE genes in cassava, Arabidopsis and sorghum. [file TPJ-105-754-s003.zip › tpj15071-sup-0003-DataS1.png]
