# Supplementary material for: Large‐scale genome‐wide association study, using historical data, identifies conserved genetic architecture of cyanogenic glucoside content in cassava (Manihot esculenta Crantz) root
Source: Plant J. 2020 Dec 18;105(3):754–70. doi: 10.1111/tpj.15071 (PMC7898387; doi:10.1111/tpj.15071)
Supplement: Supplementary file 4 — Methods S1. Proportion of variance explained by markers. Methods S2. Genome‐wide epistasis interactions. Methods S3. Cultivated and cassava progenitor differentiating loci analysis. Methods S4. KASPAR marker design and assessment. Methods S5. Candidate gene protein topology and structure prediction. Methods S6. Single point mutation prediction. Methods S7. Geographical distribution of HCN. Methods S8. GWAS in African population and joint African and Latin American analysis. [file TPJ-105-754-s004.docx]

Title: **Large scale GWAS using historical data identifies a conserved genetic architecture of cyanogenic glucoside content in cassava *(Manihot esculenta Crantz.*) root**

**Authors:** Alex C Ogbonna^1,2^, Luciano Rogerio Braatz de Andrade^3^, Ismail Y. Rabbi^4^, Lukas A. Mueller^1,2^, Eder Jorge de Oliveira^3^ and Guillaume J. Bauchet^2^

^1^ Cornell University, Ithaca, NY, USA. ^2^ Boyce Thompson Institute for Plant Research, Ithaca, NY, USA. ^3^ Embrapa Mandioca e Fruticultura, Cruz das Almas, BA - Brazil.^4^ International institute of Tropical Agriculture, Ibadan, Oyo state, Nigeria

The following **Supporting** Information is available for this article:

**Supporting Methods S1:**

**Proportion of variance explained by markers**

To determine the proportion of variance explained by the discovered loci for HCN, we used a parametric mixed model (SPMM) multiple kernel approach as previously described by Akdemir and Jannink [(2015)](https://paperpile.com/c/dtWt5D/q1ijB). Briefly, the approach incorporates the marginal variance contribution from each kernel matrix.

**Supporting Methods S2:**

**Genome-wide epistasis interaction analysis**

Genome-wide epistatic interactions between pairs of SNPs across the genome were carried out using FaST-LMM [(Lippert et al., 2011, 2013)](https://paperpile.com/c/dtWt5D/KY7Fu+11ahU), an approach that scales linearly with cohort size (sample size) in both run time and memory use. The method is based on maximum-likelihood estimates (REML for epistasis interactions) and accounts for the problem of confounding by population structure, family structure and cryptic relatedness [(Widmer et al., 2014)](https://paperpile.com/c/dtWt5D/fud5I). The Bonferroni threshold was used to test for interactions that were significant and the observed -log10(p-value) was compared against the expected using the quantile-quantile plot.

**Supporting Methods S3:**

**Cultivated and cassava progenitor differentiating loci analysis**

To assess fixed or nearly fixed loci differentiating between cultivated (*M. esculenta*) and wild cassava (*M. flabellifolia*) to investigate if cassava domestication targeted upstream or downstream genetic regulation steps of cyanide bio-synthesis. We compared differentiating loci using method earlier described by [(Bredeson et al., 2016; Wolfe et al., 2019](https://paperpile.com/c/dtWt5D/uzK0W+v71K); Ogbonna et al., 2020) using Whole-Genome sequencing HapMap II dataset [(Ramu et al. 2017)](https://paperpile.com/c/dtWt5D/lATO) and contrasted groups (cultivated and progenitors) of 5 representative accessions each based on the phylogenetic tree from Ramu et al [(Ramu et al., 2017)](https://paperpile.com/c/dtWt5D/lATO), see **Supporting Table S6**.

**Supporting Method S4:**

**Kaspar Marker Design and Assessment**

Based on association peaks, local linkage disequilibrium and allelic effect on HCN content, 6 KASP SNP markers (Supplemental table 7) were designed from available genome sequences (v6.1) including positions. Flanking regions (100 bp) were extracted for each SNP and submitted for designability following the manufacturer's recommendation (LGC genomics, Malden, MA, USA).

**Supporting Methods S5:**

**Candidate gene protein Topology and Structure Prediction**

Transmembrane protein topology of our candidate gene (Manes.16G007900) was predicted using transmembrane hidden Markov model (TMHMM, <https://services.healthtech.dtu.dk/service.php?TMHMM-2.0>) according to the method described by [(Krogh et al., 2001)](https://paperpile.com/c/dtWt5D/bgbea). The protein structure was modelled using the Phyre2 server (http://www.sbg.bio.ic.ac.uk/phyre2) following the procedure outlined in Kelley et al. [(Kelley et al. 2015)](https://paperpile.com/c/dtWt5D/120D2).Subsequent protein molecules fold stability changes (ddG) upon single point-mutations were predicted using STRUM (<https://zhanglab.ccmb.med.umich.edu/STRUM/>) following the approach outlined in Quan et al. [(Quan, Lv, and Zhang 2016)](https://paperpile.com/c/dtWt5D/JBQst).

**Supporting Methods S6:**

**Single Point Mutation Prediction**

Allele Mining and mutation prediction in whole genome resequencing data for Manes.16G007900 and Manes.16G008000 proteins. STRUM (<https://zhanglab.ccmb.med.umich.edu/STRUM/>) was used for predicting the fold stability change (ΔΔG) of protein molecules upon single-point SNP mutations. STRUM adopts a gradient boosting regression approach to train the Gibbs free-energy changes on a variety of features at different levels of sequence and structure properties. Change in free energy (ranges between -5 to 5) below zero means that the mutation causes destabilization. Mutations with sensitive stability changes can affect the motion and fluctuation of the target residues.

Point Mutation Prediction: Prediction on the SNP mutation-induced stability changes is important to protein function annotation. Wild-type amino acids are usually more stable and adoptable to the protein environments due to the long-term evolution than the new mutations. The relatively uniform stability from the wide-type amino acids when compared to the identity of the mutated amino acids should provide more information with regard to the stability changes upon new mutations [(Quan et al., 2016)](https://paperpile.com/c/dtWt5D/JBQst).

**Supporting Methods S7:**

**Geographical Distribution of HCN**

Using Tess3 [(Caye et al. 2016)](https://paperpile.com/c/dtWt5D/hHupE)and available georeferenced data for our GWAS dataset (Ogbonna et al, in press), we plotted the geospatial allele frequency distribution for the candidates associated with SNP on chromosomes 14 and 16, respectively. Individual ancestry coefficients, the proportions of an individual genome that originate from multiple ancestral gene pools, were estimated from their allelic frequency [(Frichot et al. 2014)](https://paperpile.com/c/dtWt5D/vw9ky).

**Supporting Methods S8:**

**GWAS in African Population and Joint Africa, Latin America Analysis**

To validate our findings in African cassava we sourced HCN trial experiments (228) from the cassava breeding database [cassavabase.org](https://www.cassavabase.org/). These trials were conducted by the International Institute for Tropical Agriculture (IITA) in West Africa between 1996 through 2017 across multiple locations (18) with 18,794 plots assayed for HCN. We carried out phenotypic and GWAS analysis as earlier described for Brazilian germplasm. GWAS analysis was performed on 636 unique individuals with phenotypic and genotypic information along with 53,547 SNPs. In addition, we performed joint phenotypic and GWAS analysis on African and Brazilian populations. The individuals with both phenotypic and genotypic information were 1,875 (Brazil, 1239; Africa, 636; **Supporting** Table S15) and were used for the GWAS analysis along with 17,773 common SNP loci.

We finally performed a whole genome imputation of the African-Brazilian dataset using using beagle4.0 [(Browning and Browning 2009)](https://paperpile.com/c/dtWt5D/QG6Dq)with 10 iterations, a window of 5000 markers and an overlap window of 500 markers and the HapMap as a reference panel for chromosome 16 and this dataset was used for GWAS analysis for cyanide.

**References**

[Akdemir, D. and Jannink, J.L. (2015) Locally epistatic genomic relationship matrices for genomic association and prediction, *Genetics*, 199(3), 857–871](http://paperpile.com/b/QxM6oc/2ybcI)

[Bredeson, J. V. *et al.* (2016) ‘Sequencing wild and cultivated cassava and related species reveals extensive interspecific hybridization and genetic diversity, *Nature biotechnology*, 34(5), 562–570.](http://paperpile.com/b/QxM6oc/ZfrON)

[Browning, B. L. and Browning, S. R. (2009) A unified approach to genotype imputation and haplotype-phase inference for large data sets of trios and unrelated individuals, *American journal of human genetics*, 84(2), 210–223](http://paperpile.com/b/QxM6oc/Q5W3T)

[Caye, K. *et al.* (2016) TESS3: fast inference of spatial population structure and genome scans for selection, *Molecular Ecology Resources*, 540–548. doi:](http://paperpile.com/b/QxM6oc/sdoTY) [10.1111/1755-0998.12471](http://dx.doi.org/10.1111/1755-0998.12471)

[Frichot, E. *et al.* (2014) Fast and efficient estimation of individual ancestry coefficients, *Genetics*, 196(4), pp. 973–983](http://paperpile.com/b/QxM6oc/xi8ke)

[Kelley, L. A. *et al.* (2015) The Phyre2 web portal for protein modeling, prediction and analysis, *Nature protocols*, 10(6), pp. 845–858](http://paperpile.com/b/QxM6oc/Br13U)

[Krogh, A. *et al.* (2001) Predicting transmembrane protein topology with a hidden Markov model: application to complete genomes, *Journal of molecular biology*, 305(3), pp. 567–580](http://paperpile.com/b/QxM6oc/M4tqc)

[Lippert, C. *et al.* (2011) FaST linear mixed models for genome-wide association studies, *Nature Methods*, pp. 833–835. doi:](http://paperpile.com/b/QxM6oc/Ro2Gq) [10.1038/nmeth.1681](http://dx.doi.org/10.1038/nmeth.1681)

[Lippert, C. *et al.* (2013) An exhaustive epistatic SNP association analysis on expanded Wellcome Trust data, *Scientific reports*, 3, 1099](http://paperpile.com/b/QxM6oc/bRq5C)

Ogbonna, A. C. *et al.* (2020) Comprehensive genotyping of Brazilian Cassava (Manihot esculenta Crantz) Germplasm Bank: insights into diversification and domestication, *bioRxiv* 2020.07.13.200816. <https://doi.org/10.1101/2020.07.13.200816>

[Quan, L., Lv, Q. and Zhang, Y. (2016) STRUM: structure-based prediction of protein stability changes upon single-point mutation, *Bioinformatics*, 2936–2946. doi:](http://paperpile.com/b/QxM6oc/vCau0) [10.1093/bioinformatics/btw361](http://dx.doi.org/10.1093/bioinformatics/btw361)

[Ramu, P. *et al.* (2017) Cassava haplotype map highlights fixation of deleterious mutations during clonal propagation, *Nature genetics*, 49(6), 959–963](http://paperpile.com/b/QxM6oc/eMBqj)

[Widmer, C. *et al.* (2014) Further improvements to linear mixed models for genome-wide association studies, *Scientific reports*, 4, p. 6874](http://paperpile.com/b/QxM6oc/2Jr0I)

[Wolfe, M. D. *et al.* (2019) Historical Introgressions from a Wild Relative of Modern Cassava Improved Important Traits and May Be Under Balancing Selection, *Genetics*, 213(4), 1237–1253](http://paperpile.com/b/QxM6oc/nbnSg)
