# Supplementary material for: Large‐scale genome‐wide association study, using historical data, identifies conserved genetic architecture of cyanogenic glucoside content in cassava (Manihot esculenta Crantz) root
Source: Plant J. 2020 Dec 18;105(3):754–70. doi: 10.1111/tpj.15071 (PMC7898387; doi:10.1111/tpj.15071)
Supplement: Supplementary file 5 — Appendix S1. Population structure analysis. Appendix S2. Phylogenetic tree. Appendix S3. Geographical distribution of sweet and bitter cassava. [file TPJ-105-754-s005.docx]

Title: **Large scale GWAS using historical data identifies a conserved genetic architecture of cyanogenic glucoside content in cassava *(Manihot esculenta Crantz.*) root**

**Authors:** Alex C Ogbonna^1,2^, Luciano Rogerio Braatz de Andrade^3^, Ismail Y. Rabbi^4^, Lukas A. Mueller^1,2^, Eder Jorge de Oliveira^3^ and Guillaume J. Bauchet^2^

^1^ Cornell University, Ithaca, NY, USA. ^2^ Boyce Thompson Institute for Plant Research, Ithaca, NY, USA. ^3^ Embrapa Mandioca e Fruticultura, Cruz das Almas, BA - Brazil.^4^ International institute of Tropical Agriculture, Ibadan, Oyo state, Nigeria

The following **Supporting** Information is available for this article:

**Supporting Appendix S1:**

**Population structure analysis**

Population stratification analysis was conducted on a larger population of 3354 individuals using Discriminant Analysis of Principal Components (DAPC) [(Jombart et al., 2010)](https://paperpile.com/c/dtWt5D/35mjB)and parametric Admixture [(Alexander et al., 2009)](https://paperpile.com/c/dtWt5D/wlgMb)with 5 folds cross validations for 20 K (assumed number of ancestral populations) to define the optimal number of clusters and examine patterns of relatedness and sub-ancestry among individuals in the Brazilian dataset as earlier described by Ogbonna et al., (2020).

Population structure for these materials have been extensively discussed in a parallel study by Ogbonna et al., (2020). Principal component analysis for 1,246 HCN assayed individuals with 9,686 SNPs (with Hardy-Weinberg and LD filtering) in the Brazilian germplasm shows structure patterns in our population with the first three PCs accounting for over 15.3% genetic variation.

**Supporting Appendix S2:**

**Phylogenetic tree**

Genome wide phylogenetic analysis of MATE genes in cassava, sorghum and arabidopsis suggested homology between our candidate, SbMATE2 and AT3G21690 genes. While SbMATE2 was discussed in the main text, additional discussion is provided for AT3G21690. SbMATE2 [(Darbani et al. 2016)](https://paperpile.com/c/PItHyi/SBgn) and AT3G21690 [(Liu et al. 2009)](https://paperpile.com/c/PItHyi/jmzZ) are characterized as vacuolar membrane transporters in sorghum and Arabidopsis for cyanogenic glucoside, respectively. Koh and colleague (2010), suggested that, in suspension-cultured cells, overexpression of AT3G21690 affects the vacuolar accumulation of flavonoids or affects the biosynthesis of flavonoids by unknown mechanisms. In addition, a significant increase was observed in the accumulation of putative glucosinolates and sinapate derivatives in rosette leaves of the transgenic plants of Arabidopsis. Implying that a transporter encoded by AT3G21690 may transport multiple substrates according to the cell-type dependent metabolic activities (Koh et al. 2010). Manes.16G00800 showed closer sequence homology with AT1G61890 and AT1G11670. Within the same tree cluster, AT3G59030 is characterized as TT12 [(Debeaujon et al., 2001; Marinova et al., 2007)](https://paperpile.com/c/dtWt5D/8qleI+QI1Cn)and homologue in tobacco [(Shoji et al., 2009)](https://paperpile.com/c/dtWt5D/CHbCU).

**Supporting Appendix S3:**

**Sweet and Bitter cassava geographical distribution (further discussion)**

Our finding was congruent with previous studies and presents new insights on cyanide spatial genetics. Clement and colleagues (2010), reported that bitter cassava cultivation was associated with the courses of the major Amazonian rivers, as well as the coastal areas of South America, where population densities were highest before conquest, while sweet cassava is the main crop throughout the headwaters of these same rivers in western Amazonia (Clement et al., 2010). In addition, Mckey and Beckerman (1993), reported that sweet cassava is commonly grown on a smaller scale where bitter cassava is the major crop. This may be due to the costs and benefits of toxicity, with greater benefits for large sedentary populations usually having semi-permanent fields, attracting greater pest and pathogen pressure. However, the cost is greater for smaller, more mobile populations (McKey and Beckerman 1993; Clement et al., 2010). Mckeyand Beckerman (1993), speculated that while these ideas may explain pre-conquest distributions, it is not clear if they explain current distributions of bitter and sweet cassava in Brazil. However, we found in our current dataset that the distribution of sweet and bitter cassava still reflects pre-conquest distribution and in addition, modern breeding activities in the last 40 years within Northern, Central and Southern regions of Brazil (Ogbonna et al., 2020) had created a mixed population of low, intermediate and high cyanide varieties.

**References**

[Alexander, D. H., Novembre, J. and Lange, K. (2009) Fast model-based estimation of ancestry in unrelated individuals, *Genome research*, 19(9), 1655–1664](http://paperpile.com/b/QxM6oc/VRHsH)

[Darbani, B. *et al.* (2016) The biosynthetic gene cluster for the cyanogenic glucoside dhurrin in Sorghum bicolor contains its co-expressed vacuolar MATE transporter, *Scientific reports*, 6, 37079](http://paperpile.com/b/QxM6oc/WrUg7)

[Debeaujon, I. *et al.* (2001) The TRANSPARENT TESTA12 gene of Arabidopsis encodes a multidrug secondary transporter-like protein required for flavonoid sequestration in vacuoles of the seed coat endothelium, *The Plant cell*, 13(4), pp. 853–871](http://paperpile.com/b/QxM6oc/cDymD)

[Jombart, T., Devillard, S. and Balloux, F. (2010) Discriminant analysis of principal components: a new method for the analysis of genetically structured populations, *BMC genetics*, 11, 94.](http://paperpile.com/b/QxM6oc/Wkitr)

[Liu, J. *et al.* (2009) Aluminum-activated citrate and malate transporters from the MATE and ALMT families function independently to confer Arabidopsis aluminum tolerance, *The Plant journal: for cell and molecular biology*, 57(3), 389–399](http://paperpile.com/b/QxM6oc/TmUW)

[Marinova, K. *et al.* (2007) The Arabidopsis MATE transporter TT12 acts as a vacuolar flavonoid/H+ -antiporter active in proanthocyanidin-accumulating cells of the seed coat, *The Plant cell*, 19(6), 2023–2038](http://paperpile.com/b/QxM6oc/XZRcu)

Ogbonna, A. C. *et al.* (2020) Comprehensive genotyping of Brazilian Cassava (Manihot esculenta Crantz) Germplasm Bank: insights into diversification and domestication, *bioRxiv* 2020.07.13.200816. <https://doi.org/10.1101/2020.07.13.200816>

[Shoji, T. *et al.* (2009) Multidrug and toxic compound extrusion-type transporters implicated in vacuolar sequestration of nicotine in tobacco roots, *Plant physiology*, 149(2), 708–718](http://paperpile.com/b/QxM6oc/4zav0)
